# Ultrasonic-Assisted Stir Casting of SiO_2_‑Reinforced AA7075: Microstructure, Strengthening, and Wear

**DOI:** 10.1021/acsomega.6c10661

**Published:** 2026-10-27

**Authors:** Kunkumapogu Chinna Maddaiah, Abhishek Agarwal, Nadella Anuradha, Balram Yelamasetti, Jyothula Suresh Kumar, Jyothula Sunil Kumar

**Affiliations:** † Department of Mechanical Engineering, 207621 CMR Engineering College, Kandlakoya Road, Medchal, Hyderabad, Telangana 501401, India; ‡ Department of Energy Technology, 54561 Tallinn University of Technology, Tallinn 19086, Estonia; § Department of Mechanical Engineering, 598264 MLR Institute of Technology, Dundigal Road, Medchal, Hyderabad, Telangana 500043, India; ∥ Department of Mechanical Engineering, 574362 Sreyas Institute of Engineering and Technology, Hyderabad 500068, India; ⊥ Department of Mechanical Engineering, 846037 Jawaharlal Nehru Technological University College of Engineering, Hyderabad, Kukatpally, Hyderabad, Telangana 500085, India; # Department of Mechanical Engineering, 486579 Vignan’s Nirula Institute of Technology and Science for Women, Guntur, Andhra Pradesh 522009, India

## Abstract

The present work examines the effect of SiO_2_ nanoparticle addition on the microstructure, mechanical properties, and dry-sliding wear behavior of AA7075-based metal matrix nanocomposites. Four reinforcement levels, 0.5, 1.0, 1.5, and 2.0 wt %, were investigated. AA7075 was reinforced with SiO_2_ using an ultrasonic-assisted stir-casting process. The process produced relatively uniform SiO_2_ distributions up to 1.5 wt % and a refined aluminum matrix, whereas localized particle-rich regions became evident at 2.0 wt %. The tensile strength increased from 190 ± 15 MPa for the unreinforced alloy to 255 ± 22, 310 ± 10, 360 ± 22, and 385 ± 12 MPa for composites containing 0.5, 1.0, 1.5, and 2.0 wt % SiO_2_, respectively, corresponding to strength improvements of approximately 34.2%, 63.2%, 89.5%, and 102.6%. Microhardness also increased from 86 ± 3 VHV for AA7075 to 164 ± 12 VHV for the composite containing 2.0 wt % SiO_2_. The improvements in strength and hardness are consistent with matrix refinement, interfacial load transfer, restriction of dislocation motion, and thermal-expansion-mismatch-induced dislocation strengthening. Dry-sliding wear experiments were conducted in accordance with ASTM G99 at a sliding velocity of 3.14 m s^–1^, a total sliding distance of 6 km, and applied loads of 10–50 N. The reinforced materials generally showed lower specific wear rates than the unreinforced alloy. At 50 N, the specific wear rate decreased from approximately 6900 to 4500 μm^3^ N^–1^ mm^–1^ with the addition of 2.0 wt % SiO_2_, corresponding to a decrease of approximately 34.8%. The coefficient of friction generally decreased with increasing SiO_2_ content, and the 2.0 wt % composite exhibited the lowest values within the investigated range. SEM and TEM examinations provided evidence of changes in particle distribution and microstructural features with increasing reinforcement content, while SEM examination of the worn surfaces revealed corresponding changes in wear morphology. Overall, the present findings show that the ultrasonic-assisted stir-casting route used in this study produced AA7075–SiO_2_ metal matrix nanocomposites with increased strength and hardness and generally improved dry-sliding wear behavior within the investigated composition and test range.

## Graphical abstract

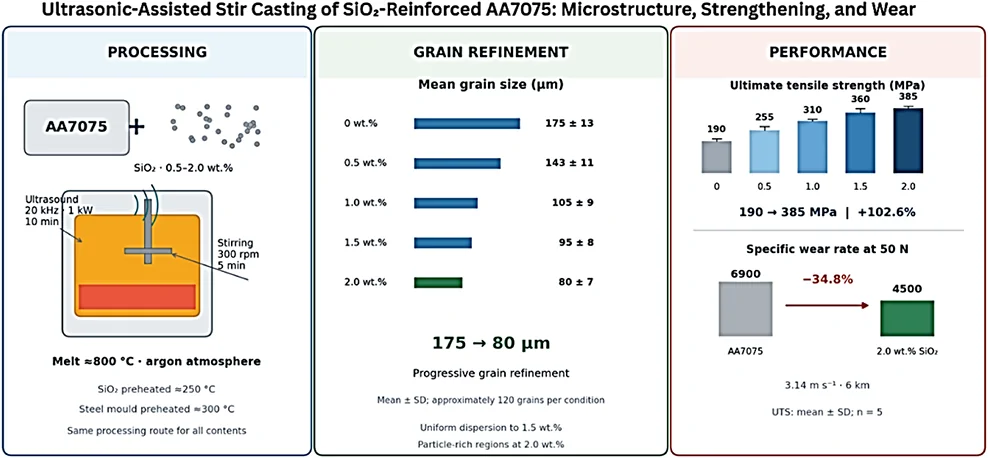

## 1 Introduction

Aluminum matrix composites (Al-MMCs) reinforced with ceramic particles are promising materials for aerospace, automotive and structural applications due to the desire for low weight materials with high specific strength and excellent wear and corrosion resistance.^1^ Appropriate particle reinforcements can be added for the purpose of microstructure modification to improve the mechanical and tribological properties of aluminum alloys.^2,3^ Al-MMCs can be manufactured by various techniques such as powder metallurgy, squeeze casting, spray deposition and liquid-metal processing depending on the desired properties and application.^4,5^ Among these methods, stir casting is the most preferred method because of its relatively simple process, low processing cost and potential for large-scale composite production.^6–8^ The addition of ceramic particles can significantly improve the strength, hardness, and wear resistance of aluminum alloys.^9^ Reinforcing particles at the nanoscale provide a high interfacial area and can provide grain refinement, effective load transfer, and dislocation strengthening, thus improving the properties of the matrix.^10^ However, incorporating nanoparticles into molten aluminum causes several processing difficulties, including poor wettability, agglomeration of particles, porosity, and nonuniform particle distribution. In an effort to overcome these limitations, ultrasonic-assisted stir casting has been used to improve particle wettability and to obtain a better dispersion of nanoparticles in the entire molten matrix.^11^ Double-staircase casting and ultrasonic vibration-assisted squeeze casting have likewise been investigated to obtain improved particle distribution and minimize processing-related defects.^12–14^ The enhanced tensile strength and ductility reported for Al-based nanocomposites have been associated mainly with grain refinement and the relatively uniform distribution of reinforcing particles.^15^ Similarly, aluminum composites reinforced with Mg_2_Si and SiC have exhibited improved strength and ductility, which have been used for the development of aluminum composites with a refinement of the eutectic phases and improved interactions between the reinforcement and the aluminum matrix.^16^ Mechanical alloying and hot extrusion are other processing techniques that have been used for the development of aluminum composites with a relatively uniform distribution of TiB_2_ particles, resulting in improved hardness and mechanical properties.^17^ The addition of ZrB_2_ to Al6061 increases the strength and hardness, and the Orowan strengthening mechanism is responsible for the increased strength observed.^18^ Also, aluminum–B_4_C composites produced through powder metallurgy have been investigated for lightweight engineering applications because of their potential combination of low density, specific strength, and wear resistance.^19,20^ Other aluminum-based nanocomposites have shown increased hardness with strengthening behavior related to Hall–Petch and Orowan mechanisms.^21^ Al–TiC nanocomposites synthesized by mechanical alloying have shown good thermal stability over a broad temperature range.^22^ In addition, the CTE (coefficient of thermal expansion) of aluminum composites can be influenced by reinforcement addition, with the thermal expansion behavior varying with reinforcement content.^23^ The mechanical response of metal matrix nanocomposites is strongly related to the size of the reinforcement, the distribution of particles, the interfacial bonding, and the chosen processing conditions. Hassan et al.^24^ reported the improvement of hardness, 0.2% yield strength, ultimate tensile strength, and ductility with the addition of nano- and submicron-sized Al_2_O_3_ atoms with Al_2_O_3_ particles/nanoparticles. The improvements were explained by grain refinement, reduced porosity, and better integrity at the matrix-particle interface. It has been reviewed in the past that, for the processing of MMCs, the appropriate choice of fabrication techniques and control of the matrix-reinforcement interface are the most important factors in achieving the desired composite properties.^25,26^ Zhang and Chen^27^ investigated the contributions of the load-bearing, dislocation-density, and Orowan strengthening mechanisms to the strength of particulate-reinforced metal matrix nanocomposites. Their analysis showed that the orowan strengthening can make a significant contribution to the overall strengthening and that its effect generally increases with decreasing nanoparticle size. However, it was found that the orowan contribution decreased dramatically when the particle size was below a critical value related to the Burgers vector of the matrix dislocations.^28^ Ultrasonic treatment can promote particle deagglomeration, improve reinforcement wettability, and enhance particle distribution in aluminum melts through cavitation and acoustic streaming effects.^29,30^ The mechanical response of aluminum composites is also influenced by grain refinement and strengthening mechanisms associated with reinforcement addition. For example, graphene-reinforced aluminum composites processed by high-pressure torsion have demonstrated substantial grain refinement and improved mechanical properties, which were associated with load transfer, grain refinement, and Orowan strengthening.^31–33^ In AA7075 and other Al–Mg–Zn–Cu alloys, heat treatment and thermomechanical processing can further modify the mechanical response through precipitation and dislocation strengthening associated with nanoscale strengthening phases.^34,35^ More broadly, the processing route, reinforcement characteristics, and matrix–particle interactions are important factors governing the balance between mechanical performance and component weight in metal matrix composites.^36^ Aluminum-based metal matrix composites have therefore attracted considerable interest for lightweight structural applications.^37–39^ Their properties depend strongly on reinforcement type, particle size, reinforcement fraction, and particle distribution. Common ceramic reinforcements investigated for aluminum matrices include Al_2_O_3_, TiC, B_4_C, SiC, WC, TiO_2_, ZrO_2_, SiO_2_, and Si_3_N_4_.^40^

Previous investigations of AA7075-based composites have reported improvements in mechanical and tribological properties following ceramic reinforcement.^41–44^ Hybrid AA7075 composites containing nano-Al_2_O_3_ and SiC showed improved strength and ductility associated with grain refinement and relatively uniform nanoparticle dispersion.^45^ Huda et al.^40^ reported increases in tensile strength, yield strength, hardness, and wear resistance in stir-cast AA7075–Al_2_O_3_ composites with increasing Al_2_O_3_ content. Other studies have similarly reported enhanced hardness and wear performance following Al_2_O_3_ addition to AA7075.^46^ Hybrid ceramic and carbon-based reinforcement systems have also demonstrated changes in strength, hardness, and wear behavior of aluminum matrix composites.^47,48^ In AA7075–TiB_2_ nanocomposites, reinforcement addition was reported to influence the coefficient of friction and overall tribological response, with friction behavior being related to surface hardness and material transfer during sliding.^49–51^ Further studies on TiB_2_-reinforced aluminum alloys have shown that adequate reinforcement distribution can contribute to improvements in hardness, strength, and wear resistance.^52–54^

Although ceramic reinforcement has been widely investigated in AA7075-based composites, the combined relationship between low SiO_2_ additions, particle distribution, strengthening behavior, and dry-sliding response remains insufficiently established for the 0.5–2.0 wt % composition range. Previous studies have mainly considered other ceramic or hybrid reinforcements, whereas the effect of increasing nanoscale SiO_2_ content on the transition from relatively uniform dispersion to localized clustering, and the corresponding changes in strength, ductility, friction, and wear, requires further examination. The present study therefore investigates AA7075 containing 0.5–2.0 wt % SiO_2_ produced using a fixed ultrasonic-assisted stir-casting route. Microstructural characterization, tensile testing, hardness measurements, dry-sliding wear tests, and analytical strengthening calculations are combined to relate reinforcement content and particle distribution to the measured mechanical and tribological response. Particular attention is given to the increasing tendency for particle clustering at higher SiO_2_ contents and its implications for the effectiveness of the proposed strengthening mechanisms.

## 2 Experimentation-Methods and Materials

In the present work, AA7075 aluminum alloy was selected as the matrix material and procured from Alloys and Vision Castings, Telangana, India. Owing to its high specific strength and favorable mechanical properties, AA7075 is widely considered for lightweight structural applications in the aerospace and transportation sectors, including wheels, brake calipers, pistons, and rocker arms. The SiO_2_ nanoparticles were procured from Sigma-Aldrich. According to the supplier specifications, the nanopowder had a nominal average particle size of approximately 20 nm, with a reported particle-size range of 10–30 nm and a near-spherical to irregular morphology. The reported purity was approximately 99.5% or higher, while the specific surface area was 150–250 m^2^ g^–1^. These values represent the nominal supplier specifications and were not independently measured in the present study; therefore, a particle size of 20 nm was adopted as the representative value for the strengthening calculations.^55,56^ Independent particle-size distribution measurements, BET surface-area analysis, and TEM characterization of the as-received SiO_2_ powder were not performed. Instead, TEM/SAED analysis was conducted on the fabricated AA7075–SiO_2_ composites to evaluate reinforcement distribution, particle clustering, and particle–matrix interactions following ultrasonic-assisted processing. Compared with AA7075, SiO_2_ has a lower density and considerably lower coefficient of thermal expansion, and the resulting thermal-expansion mismatch can generate additional dislocations during solidification and cooling, thereby contributing to thermal-mismatch strengthening. Accordingly, the processing parameters were selected to facilitate effective incorporation and dispersion of the SiO_2_ nanoparticles within the molten AA7075 matrix.

The matrix material was AA7075 alloy with SiO_2_ nanoparticles addition of 0.5, 1.0, 1.5, and 2.0 wt %. The AA7075 alloy was heated to about 800 °C to ensure good fluidity of the melt during the incorporation and mixing of SiO_2_ nanoparticles. The SiO_2_ powder was preheated to about 250 °C before addition, which reduced the temperature difference between the reinforcement and molten alloy and thus facilitated the addition to the melt. The nanoparticles were initially mixed by mechanical stirring at 300 rpm for 5 min, followed by ultrasonic treatment at 20 kHz and 1 kW for 10 min. These processing conditions were selected based on the melt-processing requirements of AA7075 and previously reported ultrasonic-assisted processing routes for aluminum-based composites.^57,58^ The selected conditions were used as a fixed processing route for all reinforcement levels; no separate optimization study of the processing parameters was performed. Before casting, the steel mold was preheated to 300 °C to minimize thermal shock and facilitate more controlled solidification of the composite. The procedure was performed in an argon atmosphere so as to reduce the unwanted interaction of the molten alloy with the ambient environment. After ultrasonic treatment the melt was poured into a steel mold of dimensions 150 × 150 × 10 mm. The combined mechanical stirring and ultrasonic treatment constituted the processing route used to incorporate and distribute the SiO_2_ particles in the AA7075 melt.

## 3 Results and Discussion

### 3.1 Microstructural Characterization of AA7075–SiO_2_ Nanocomposites

Figures 1 and 2 present SEM images and EDS elemental maps of the as-cast AA7075 alloy and the AA7075–1.0 wt % SiO_2_ nanocomposite, respectively. The maps show the distribution of the elements identified in the selected regions. For the unreinforced alloy, the maps include Al, Zn, Mg, Cu, Si, Fe, Cr and Mn. For the composite, the maps include Al, Si, O, Mg, Zn, Cu, Fe and Cr. The additional maps provide information on the local distribution of the alloying elements and the elements detected in the reinforced material. The EDS measurements represent the selected analysis regions and should not be interpreted as measurements of bulk composition. The Si signal detected in the unreinforced alloy cannot be attributed to the addition of SiO_2_, because no SiO_2_ reinforcement was added to that specimen. In the composite, the local Si signal may reflect the composition of the analyzed region, including SiO_2_-containing areas, but the elemental maps alone cannot quantify the contribution from the reinforcement. Independent bulk chemical analysis was not performed in this study; therefore, the EDS results are used to discuss local elemental distributions and are not used to establish the bulk composition or identify specific intermetallic phases.

**Figure 1:**
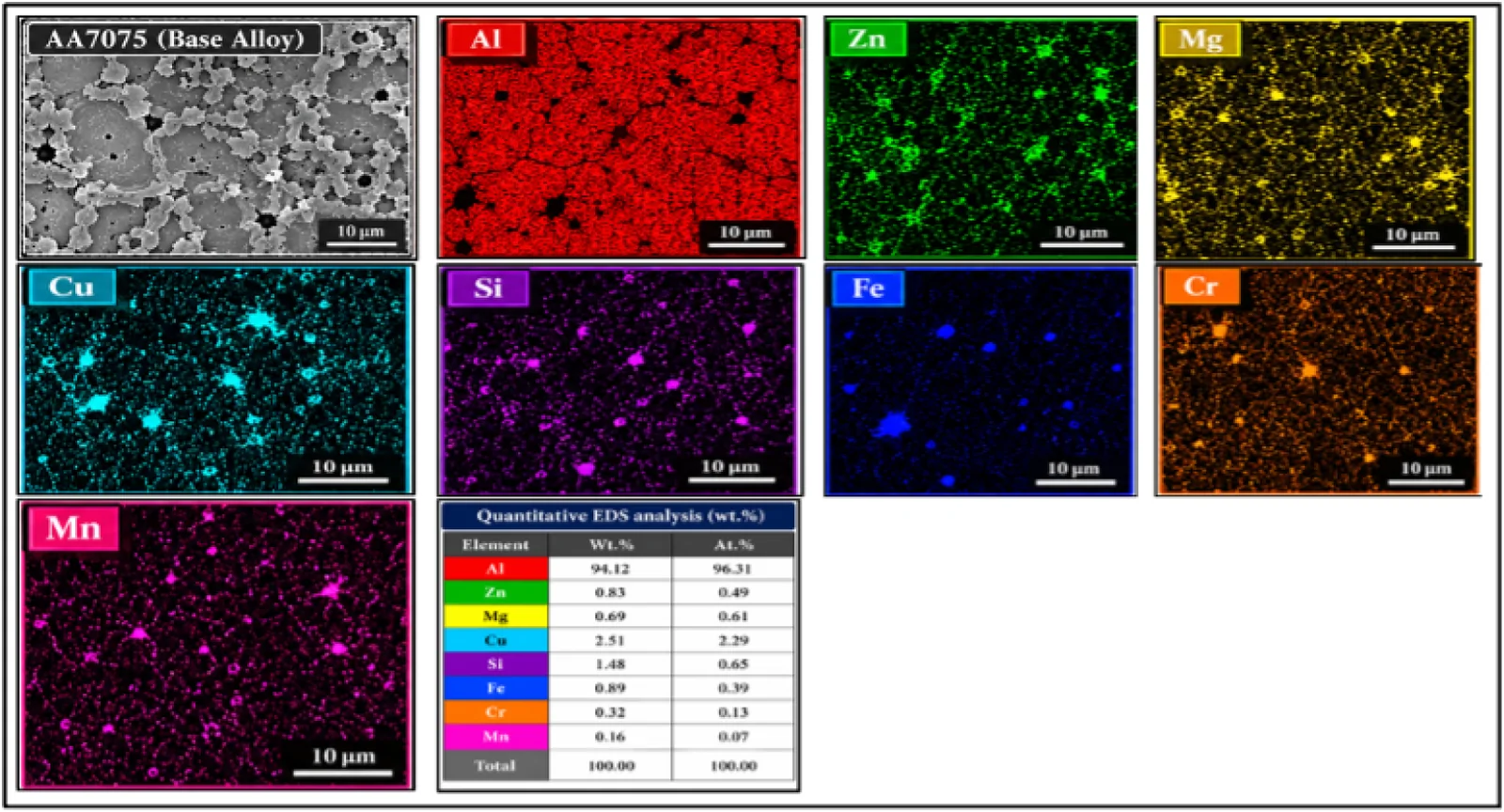
SEM micrograph and EDS elemental distribution maps of Al, Zn, Mg, Cu, Si, Fe, Cr, and Mn in the as-cast AA7075 alloy.

**Figure 2:**
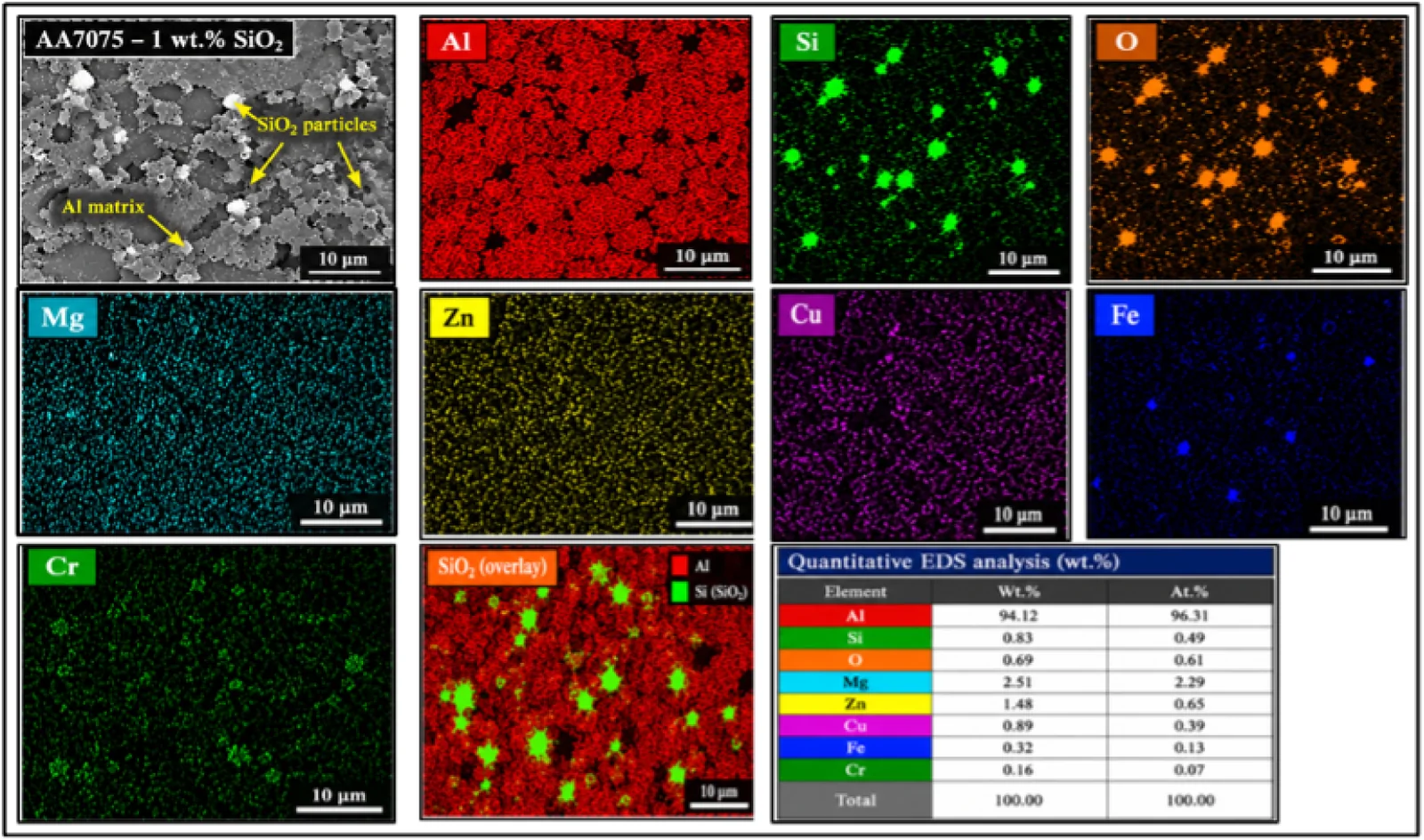
SEM micrograph and EDS elemental distribution maps of Al, Si, O, Mg, Zn, Cu, Fe, and Cr in the AA7075–1.0 wt % SiO_2_ nanocomposite.

The AA7075 designation is based on the commercially supplied starting material; an independent bulk chemical analysis was not performed in the present study and is identified as a limitation. SEM image and elemental mapping results of the AA7075–SiO_2_ nanocomposite are shown in Figure 2. The BSE image reveals several bright, particle-rich regions dispersed within the aluminum matrix, with a few localized clusters near grain and interdendritic regions. These bright features exhibit higher average atomic-number contrast than the surrounding matrix. However, because localized point analysis was not performed specifically on these regions, no definitive phase assignment is made. The corresponding elemental maps show an Al-rich matrix with localized Si- and O-rich regions in the composite. The colocalized Si and O signals are consistent with silica-containing regions; however, EDS mapping alone cannot establish the local SiO_2_ concentration or identify a specific phase. A local Si signal is also visible in the unreinforced alloy, but the available data do not establish its precise origin. As the quantitative EDS values could not be independently verified, numerical selected-area compositions are not reported. Independent bulk chemical analysis was not performed; therefore, no conclusion is drawn about the bulk silicon content or the amount of SiO_2_ incorporated into the composite.

The SEM–EDS maps are interpreted qualitatively to illustrate the local distribution of detected elements within the selected microscopic regions. They do not provide a verified quantitative comparison of silicon content between the specimens and should not be interpreted as bulk-composition measurements. The observations also do not establish the formation of specific intermetallic phases. Because a conventional stir-casting control without ultrasonic treatment was not examined, the independent effect of ultrasonic treatment on particle dispersion cannot be separated from the effect of SiO_2_ addition.

### 3.2 Optimal Microstructure and SEM Images

Microscopic characterization of AA7075 and AA7075–SiO_2_ nanocomposites was performed by optical microscopy and scanning electron microscopy on specimens prepared to dimension of 10 × 10 × 10 mm. After careful sectioning, progressive grinding and final polishing, the specimens were examined by conventional metallographic preparation. The optical and SEM micrographs presented in Figures 3 and 4 show the evolution of the AA7075 matrix as a function of SiO_2_ content. The unreinforced AA7075 exhibits relatively coarse α-Al grains with well-defined grain boundaries and interdendritic regions. The 0.5 wt % SiO_2_ condition exhibited finer matrix features and relatively uniform reinforcement distribution compared with the unreinforced condition. The microstructures are finer and more uniform with the addition of SiO_2_ up to 1.0 and 1.5 wt %. The 1.5 wt % composite shows a very uniform distribution of the particles.^59^ The observed refinement and particle distribution characterize the composites produced by the ultrasonic-assisted stir-casting route. The present experiments do not include a nonultrasonically processed control, and therefore the individual contribution of ultrasonic cavitation cannot be separated. The matrix still has a refined grain structure at 2.0 wt % SiO_2_; however, the SEM micrograph shows localized particle-rich regions and agglomerates. This indicates that the higher amount of reinforcement increases particle–particle interaction and the tendency to clustering. The OM and SEM observations show progressively finer matrix features and relatively uniform particle distribution up to 1.5 wt % SiO_2_ within the processing route used in this study. The dispersion was relatively uniform up to 1.5 wt % SiO_2_, whereas localized agglomeration became more evident at 2.0 wt % SiO_2_. The refined grain structure and relatively uniform particle distribution observed in the composites are consistent with strengthening through increased resistance to dislocation motion and load transfer between the matrix and reinforcement. Conversely, agglomerated regions can serve as local stress-concentration sites, restricting the possibilities for further mechanical performance enhancement.^60^

**Figure 3:**
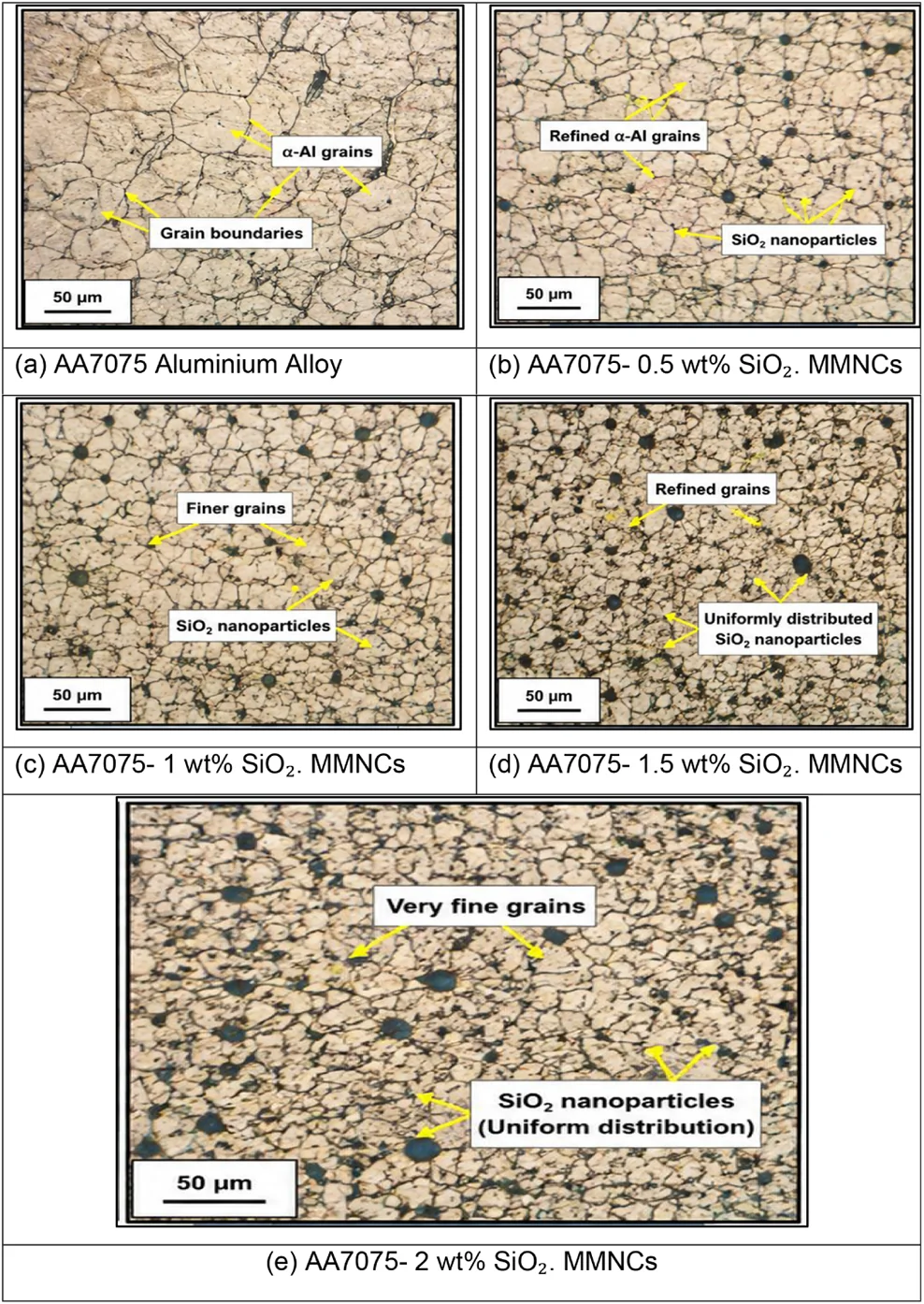
(a–e) Optical micrographs of AA7075 and AA7075–SiO_2_ nanocomposites containing 0, 0.5, 1.0, 1.5, and 2.0 wt % SiO_2_.

**Figure 4:**
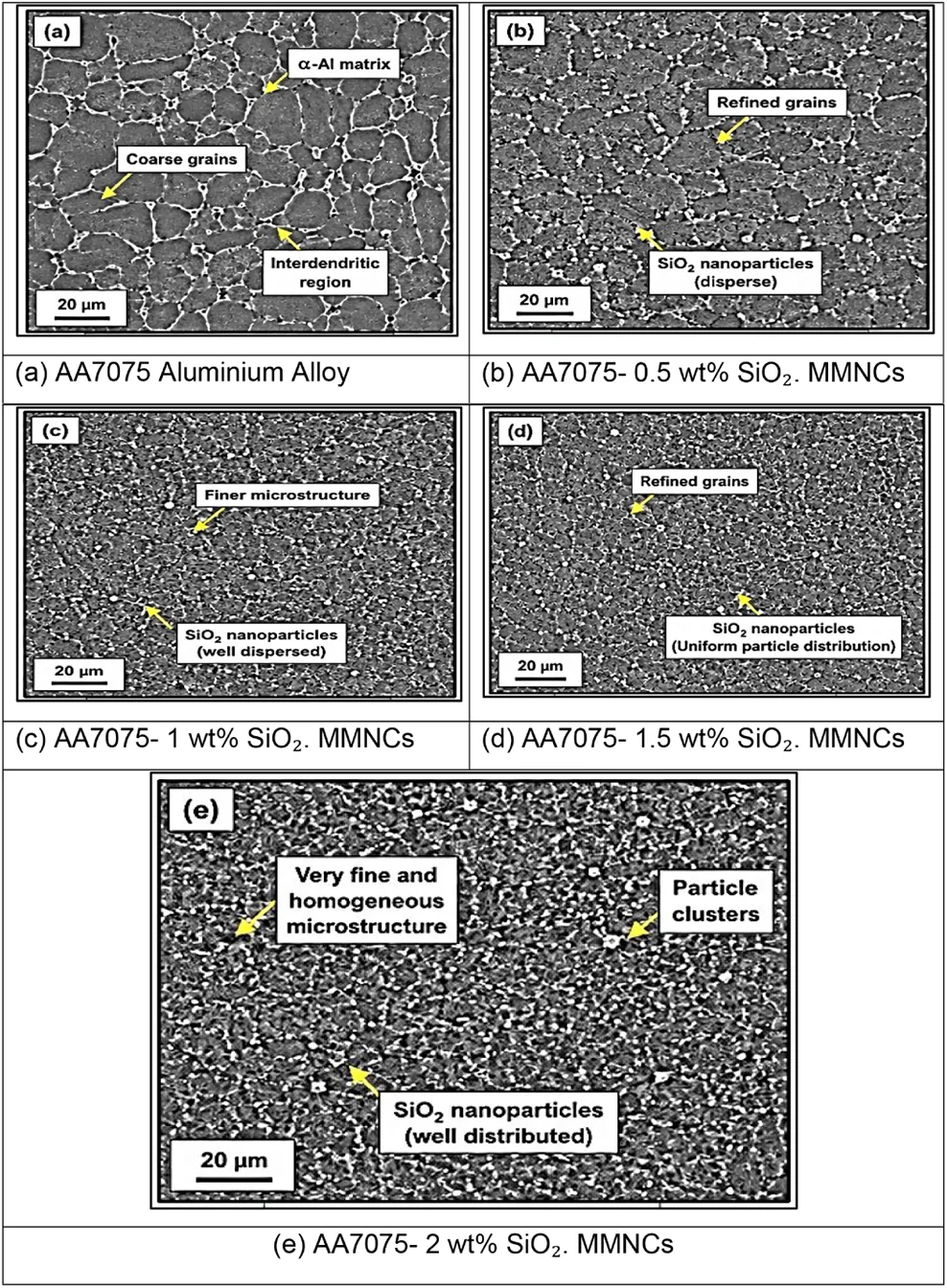
(a–e) SEM micrographs of the fabricated MMNCs: (a) AA7075, (b) AA7075–0.5 wt % SiO_2_, (c) AA7075–1.0 wt % SiO_2_, (d) AA7075–1.5 wt % SiO_2_, and (e) AA7075–2.0 wt % SiO_2_.

### 3.3 Tensile Properties of AA7075–SiO_2_ Nanocomposites

The AA7075 alloy and AA7075–SiO_2_ nanocomposites (tensile tests were performed according to the ASTM E8/E8M standard) have been prepared from as-cast materials. The tests were performed at room temperature on a programmable universal testing machine (FIE UTE100) at a constant crosshead speed of 1 mm min^–1^. For each material composition, five independent tensile specimens (n = 5) were tested under identical conditions in accordance with ASTM E8/E8M. The tensile strength increased progressively across the investigated SiO_2_ reinforcement levels, as shown in Figure 5a,b. The observed strengthening is consistent with matrix refinement, interfacial load transfer, restriction of dislocation motion, and thermal-expansion-mismatch-induced dislocation strengthening. The UTS of the base alloy was 190 ± 15 MPa, and the UTS of the composites with 0.5, 1.0, 1.5, and 2.0 wt % SiO_2_ were 255 ± 22, 310 ± 10, 360 ± 22, and 385 ± 12 MPa, respectively. These values correspond to improvements of about 34.2, 63.2, 89.5, and 102.6%, respectively, when compared to the unreinforced matrix. The reported UTS values of 190 ± 15, 255 ± 22, 310 ± 10, 360 ± 22, and 385 ± 12 MPa for AA7075, 0.5, 1.0, 1.5, and 2.0 wt % SiO_2_, respectively, are presented as mean ± standard deviation. Yield strength also showed a similar trend, where the 2.0 wt % SiO_2_ composite increased by ∼171.9% compared to the AA7075 alloy. The improvement in tensile properties can be associated with matrix grain refinement, load transfer from the aluminum matrix to the SiO_2_ particles, resistance to dislocation motion, and additional dislocations generated by the difference in thermal expansion between AA7075 and SiO_2_. The stress–strain behavior also indicates that the composites are able to withstand higher stress with an increase in SiO_2_ content, i.e., higher resistance to plastic deformation.^61,62^ The standard deviations indicate the scatter among the measured tensile results, with somewhat greater variation for the 0.5 and 1.5 wt % compositions. The composite containing 2.0 wt % SiO_2_ presented the highest UTS. The observed reinforcement-dependent strengthening is broadly consistent with previous studies of reinforced Al7075-based composites, where ceramic or hybrid reinforcements have been associated with increased hardness, tensile strength, and wear resistance through load transfer, matrix refinement, and restriction of dislocation motion.^57,63^ However, the reinforcement systems and processing conditions in these studies differ from those used here. Therefore, these comparisons provide context for the present reinforcement-dependent behavior but do not allow the independent contribution of ultrasonication to be established. Nevertheless, the microstructural observation also revealed a localized agglomeration of nanoparticles for this reinforcement content. Such particle-rich regions can induce a local stress concentration and indicate that the material is approaching a critical reinforcement content, above which the further addition of SiO_2_ can reduce the uniformity of dispersion, impair the interfacial integrity, and adversely affect the mechanical response. The 2.0 wt % SiO_2_-reinforced composite showed the highest tensile strength compared to the other reinforcements studied. The observed agglomeration underlines the importance of controlling the dispersion of nanoparticles at higher reinforcement contents.

**Figure 5:**
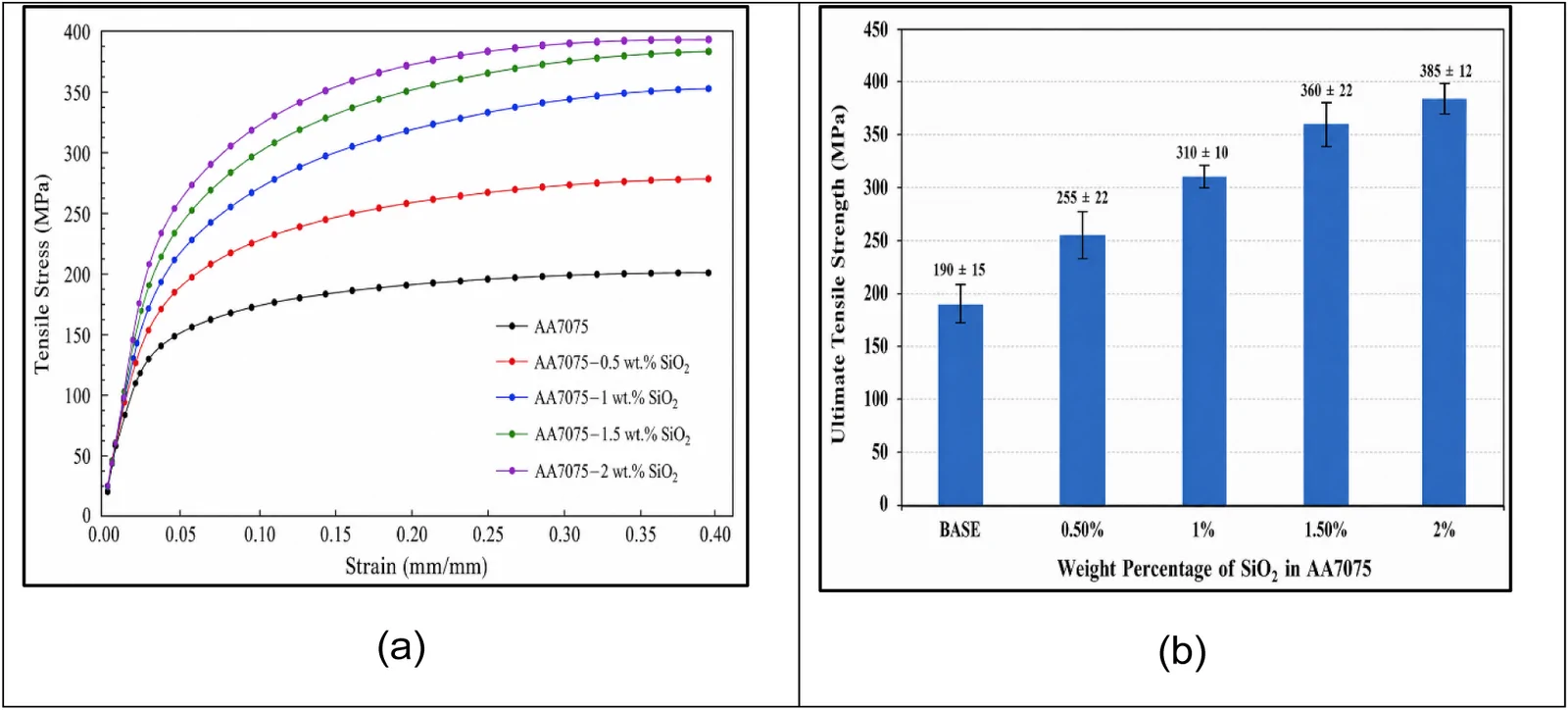
(a) Representative stress–strain curves and (b) ultimate tensile strength of AA7075–SiO_2_ nanocomposites.

### 3.4 Microhardness, Density, and Porosity of AA7075–SiO_2_ Nano MMNCs

The effect of adding SiO_2_ nanoparticles on the physical and mechanical properties of AA7075-based metal matrix nanocomposites was studied by measuring the density, porosity, and Vickers microhardness. Figure 6a shows the comparison between the theoretical and experimentally measured densities of the investigated materials. The theoretical density of unreinforced AA7075 alloy was 2.830 g cm^–3^, and it increased progressively to 2.835, 2.840, 2.846, and 2.851 g cm^–3^ with the addition of 0.5, 1.0, 1.5, and 2.0 wt % SiO_2_, respectively, calculated from the rule of mixtures. The experimental densities were 2.822 ± 0.003, 2.826 ± 0.003, 2.832 ± 0.002, 2.839 ± 0.002, and 2.841 ± 0.003 g cm^–3^, respectively. The theoretical density (ρth) was obtained using the rule of mixtures, whereas the experimental density (ρexp) was determined from the fabricated specimens. The percentage porosity was calculated from the density deficit using

$$\mathrm{Porosity}\;\left(\%\right)=\frac{\rho_{\mathrm{th}}-\rho_{\exp}}{\rho_{\mathrm{th}}}\times 100$$

**Figure 6:**
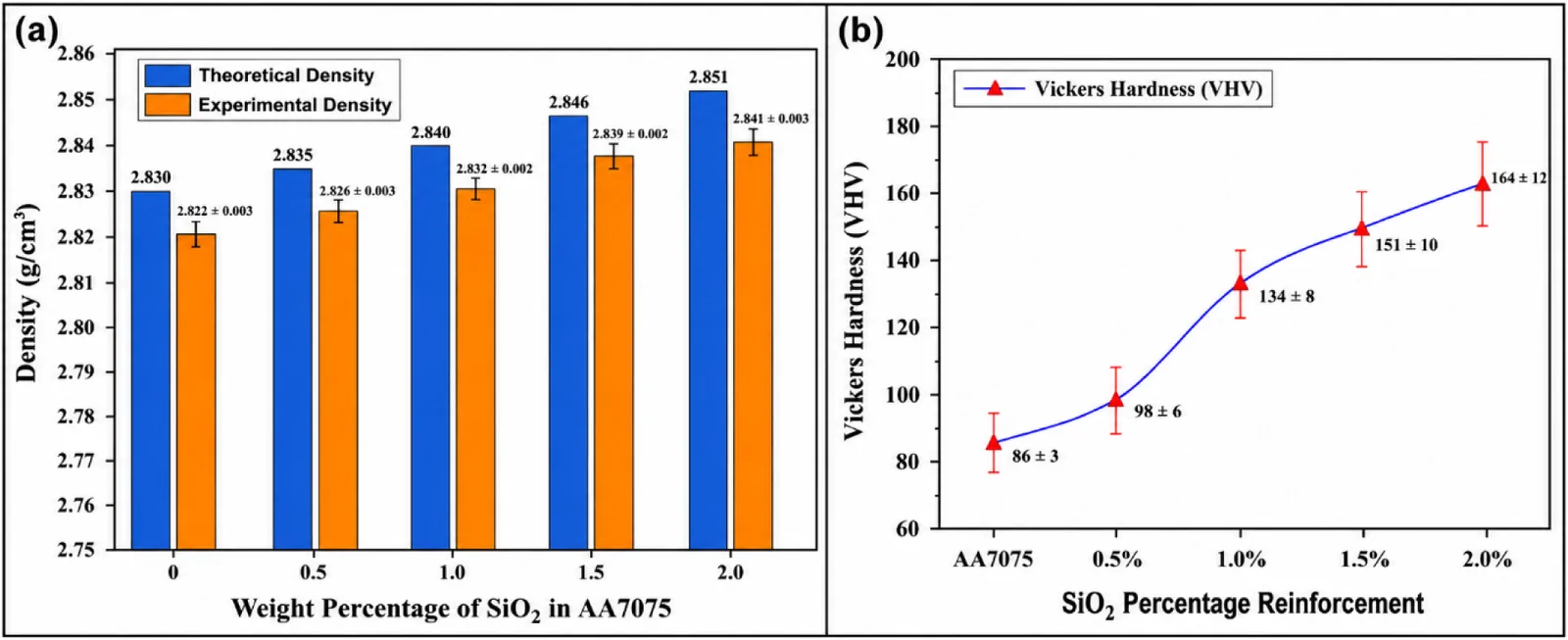
(a) Comparison of the theoretical and experimentally measured densities of AA7075–SiO_2_ nanocomposites as a function of SiO_2_ reinforcement content and (b) Vickers microhardness of AA7075–SiO_2_ nanocomposites.

For the unreinforced AA7075 alloy, ρth = 2.830 g cm^–3^ and ρexp = 2.822 g cm^–3^, corresponding to approximately 0.283% porosity. The corresponding calculated porosity values for the 0.5, 1.0, 1.5, and 2.0 wt % SiO_2_ composites were approximately 0.317%, 0.282%, 0.246% and 0.351%, respectively. These values represent density-derived porosity rather than direct microscopic porosity measurements. The small differences between theoretical and experimental density indicate a limited density deficit in the fabricated materials. The gradual rise in density with increasing SiO_2_ concentration is mainly related to the contribution of the incorporated ceramic phase to the overall mass and volume fraction of the composite. The relatively small difference between theoretical and measured densities further suggests that the fabricated materials contained only a limited amount of internal porosity. The measured density differences indicate a limited level of density deficit relative to the theoretical values. Because no conventional stir-cast control without ultrasonic treatment was examined, the present data cannot be used to quantify any independent contribution from ultrasonic processing. In addition, the dispersed SiO_2_ nanoparticles may provide heterogeneous nucleation sites during solidification, which can contribute to refinement of the α-Al matrix. The effect of SiO_2_ addition on microhardness was determined using an ECONOMET VH 1MD Vickers microhardness tester at room temperature. According to ASTM E92, five indentations were made for each composition to get the average value, and a 200 g load was applied for a dwell time of 15 s. The hardness of the unreinforced AA7075 alloy was 86 ± 3 VHV (Figure 6b). The hardness increased with increasing SiO_2_ content to 98 ± 6, 134 ± 8, 151 ± 10, and 164 ± 12 VHV for 0.5, 1.0, 1.5, and 2.0 wt % SiO_2_, respectively. This enhancement is attributed to the intrinsic hardness of the SiO_2_ phase, the refinement of the microstructure of the matrix, the inhibition of the dislocation movement, the increased resistance to localized deformation, and the load transfer between the aluminum matrix and the dispersed nanoparticles. Therefore, the combined density, porosity, and hardness results show that the composite properties are clearly dependent on SiO_2_ content, with the 2.0 wt % SiO_2_ specimen having the highest measured density and microhardness of the compositions studied.

Although localized clustering and the highest measured porosity were observed at 2.0 wt % SiO_2_, the larger reinforcement content also increased the number of hard particles and matrix-particle interfaces available for strengthening. Within the analytical model, the calculated Orowan contribution was approximately 26.7 MPa, while thermal-expansion-mismatch dislocation strengthening reached approximately 138 MPa, the largest calculated contribution among the mechanisms considered. Together with load transfer and grain refinement, these contributions were sufficient to maintain the measured strengthening response despite localized defects. The 2.0 wt % SiO_2_ composite therefore exhibited the highest measured UTS (385 ± 12 MPa) and hardness (164 ± 12 VHV) within the investigated composition range. Nevertheless, clustering and porosity can reduce reinforcement efficiency by introducing local stress concentrations and nonuniform load transfer, and further SiO_2_ addition should not be assumed to produce additional improvement.

### 3.5 Density-Derived Porosity of AA7075–SiO_2_ MMNCs

Figure 7 shows the variation in density-derived porosity with increasing SiO_2_ reinforcement content. The porosity percentage was determined from the difference between the theoretical density, *ρ*_th_ calculated using the rule of mixtures, and the experimentally measured density, *ρ*_exp_, according to

$$P\;\left(\%\right)=\frac{\rho_{\mathrm{th}}-\rho_{\exp}}{\rho_{\mathrm{th}}}\times 100$$

**Figure 7:**
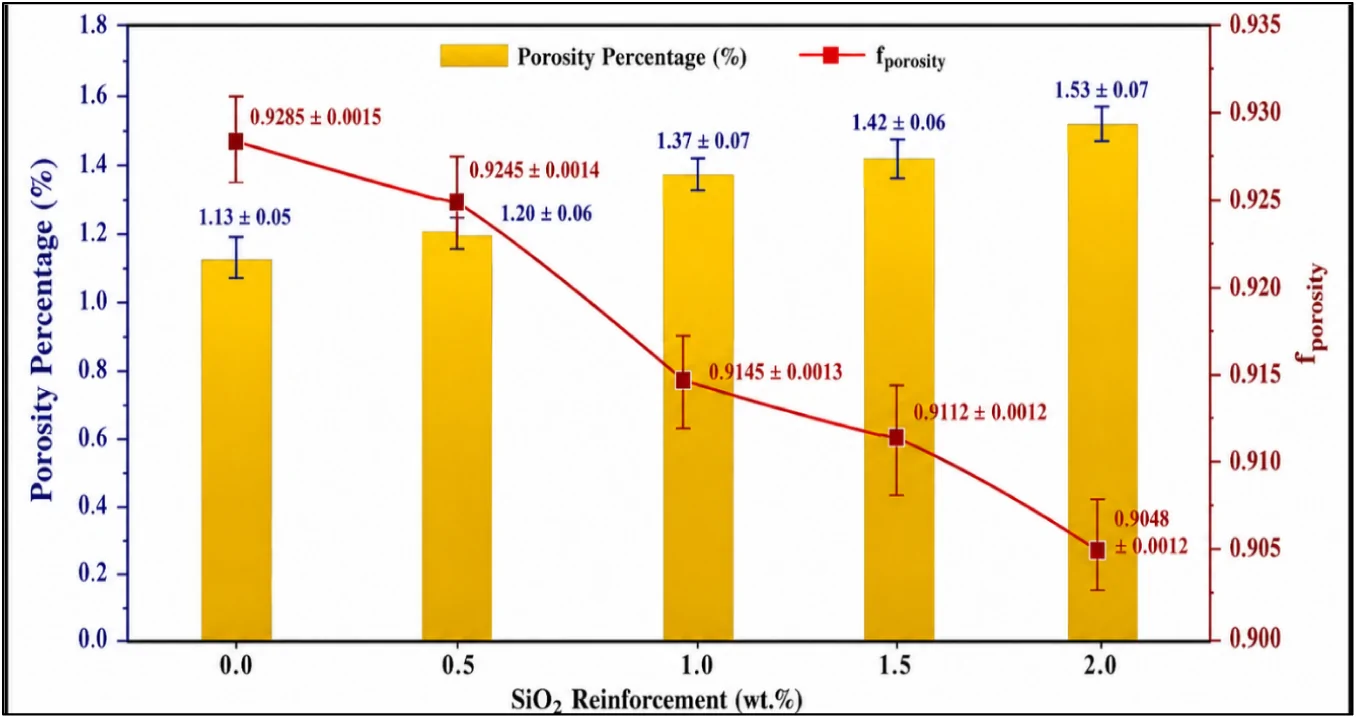
Density-derived porosity of AA7075 and AA7075–SiO_2_ nanocomposites as a function of SiO_2_ reinforcement content.

The calculated porosity values were approximately 0.283%, 0.317%, 0.282%, 0.246%, and 0.351% for AA7075 and 0.5, 1.0, 1.5, and 2.0 wt % SiO_2_, respectively. These values represent density-derived estimates rather than direct measurements of individual pores. The relatively small density deficit indicates that the specimens remained close to their theoretical densities. The slightly higher value at 2.0 wt % SiO_2_ is consistent with the localized particle-rich regions observed in the microstructural analysis. Such regions may contribute to local void formation through incomplete wetting, particle accumulation, or gas entrapment during processing and solidification.^64^

### 3.6 Ductility of AA7075–SiO_2_ Nanocomposites

The percentage elongation of the AA7075–SiO_2_ nanocomposites decreases with increasing SiO_2_ content, as shown in Figure 8. The maximum elongation of unreinforced AA7075 alloy was found to be 11.2 ± 1.5%, whereas the addition of 0.5 wt % SiO_2_ reduced the value to 10.0 ± 0.7%. With further reinforcement, the elongation drops to 9.7 ± 0.7% at 1.0 wt % SiO_2_ and further decreases to 9.2 ± 0.4% at 1.5 wt % SiO_2_. The AA7075–2.0 wt % SiO_2_ composite showed the lowest elongation of 8.6 ± 0.8%. This increasing loss of ductility is attributed to larger contents of rigid SiO_2_ nanoparticles, which impede the plastic flow in the aluminum matrix and inhibit the motion of dislocations.^65^ At large reinforcement, the particle–particle interaction and localized clustering can further restrict the matrix from accommodating uniform plastic strain. Therefore, the addition of SiO_2_ increases the strength and hardness but decreases the deformation ability of the composite. Thus, the trend obtained shows the compromise between strength and ductility, and the composite of 2.0 wt % SiO_2_ has the lowest elongation of the studied compositions.

**Figure 8:**
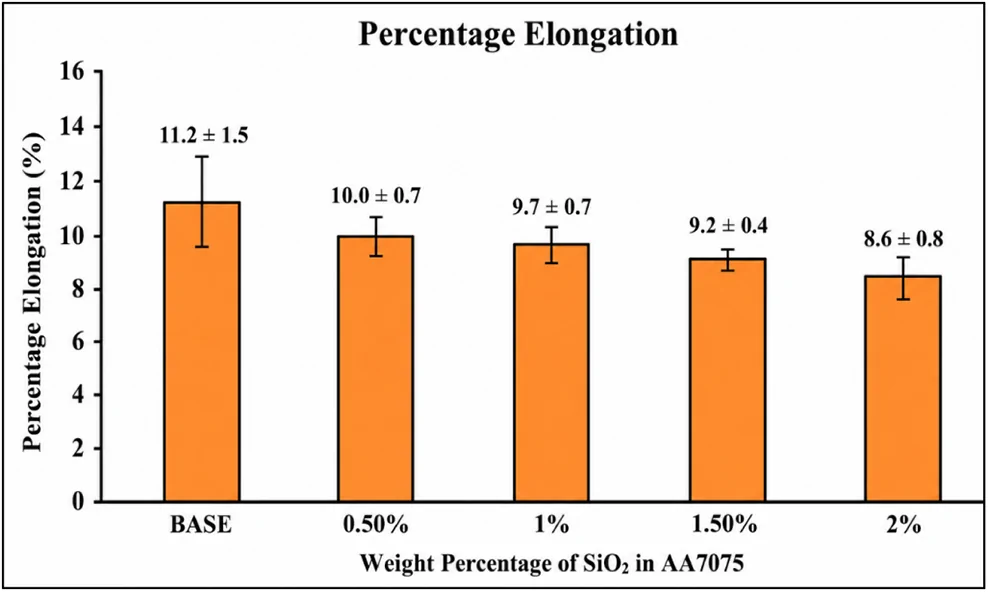
AA7075–SiO_2_ MMNCs ‘percentage elongation.

### 3.7 Tensile Fracture SEM Microstructure of AA7075–SiO_2_ MMNCs

SEM micrographs of tensile fracture reveal a gradual change in the fracture features of AA7075 with an increase in SiO_2_ reinforcement content as shown in Figure 9a–e. The unreinforced alloy shows large and deep dimples, distinct tearing ridges, and abundant microvoid coalescence, signifying the high amount of localized plastic deformation before fracture. Besides the presence of increased heterogeneity and the increased number of dimples in the fracture surface after the addition of 0.5 wt % SiO_2_, localized debonding between the particles and the matrix is also visible. This shows that the interfacial bonding is not uniform at this level of reinforcement.^66^ For a composite with 1.0 wt % SiO_2_, the dimple size is smaller. Fractographs show a finer and more uniform distribution of fracture features. The more efficient transfer of stress from the AA7075 matrix to the reinforcement is observed with better bonding between the particles and matrix. The fracture surface was relatively homogeneous with the increasing SiO_2_ content up to 1.5 wt % and consisted of fine and shallow dimples, well-bonded nanoparticles, and obvious crack-deflection features. The features indicate that the uniformly distributed nanoparticles restrict the localized plastic deformation and alter the path of crack propagation, which contributes to the improved tensile response. In contrast, the sample reinforced with 2.0 wt % SiO_2_ exhibits localized clustering of nanoparticles, particle pull-out, and shallow dimples. The fracture morphology still maintains a relatively refined structure. However, the particle agglomeration and pull-out indicate a decrease in the uniformity of interfacial bonding at the highest reinforcement content. Such clustered regions can act as preferential sites for stress concentration and crack initiation and therefore weaken further improvement of the tensile performance. To summarize, the fractographic analysis reveals a progressive evolution of the fracture morphology and an improved particle–matrix interaction for up to 1.5 wt % SiO_2_, while localized agglomeration and interfacial defects are more pronounced at 2.0 wt % SiO_2_, indicating that the higher reinforcement content increased microstructural heterogeneity at the fracture surface.

**Figure 9:**
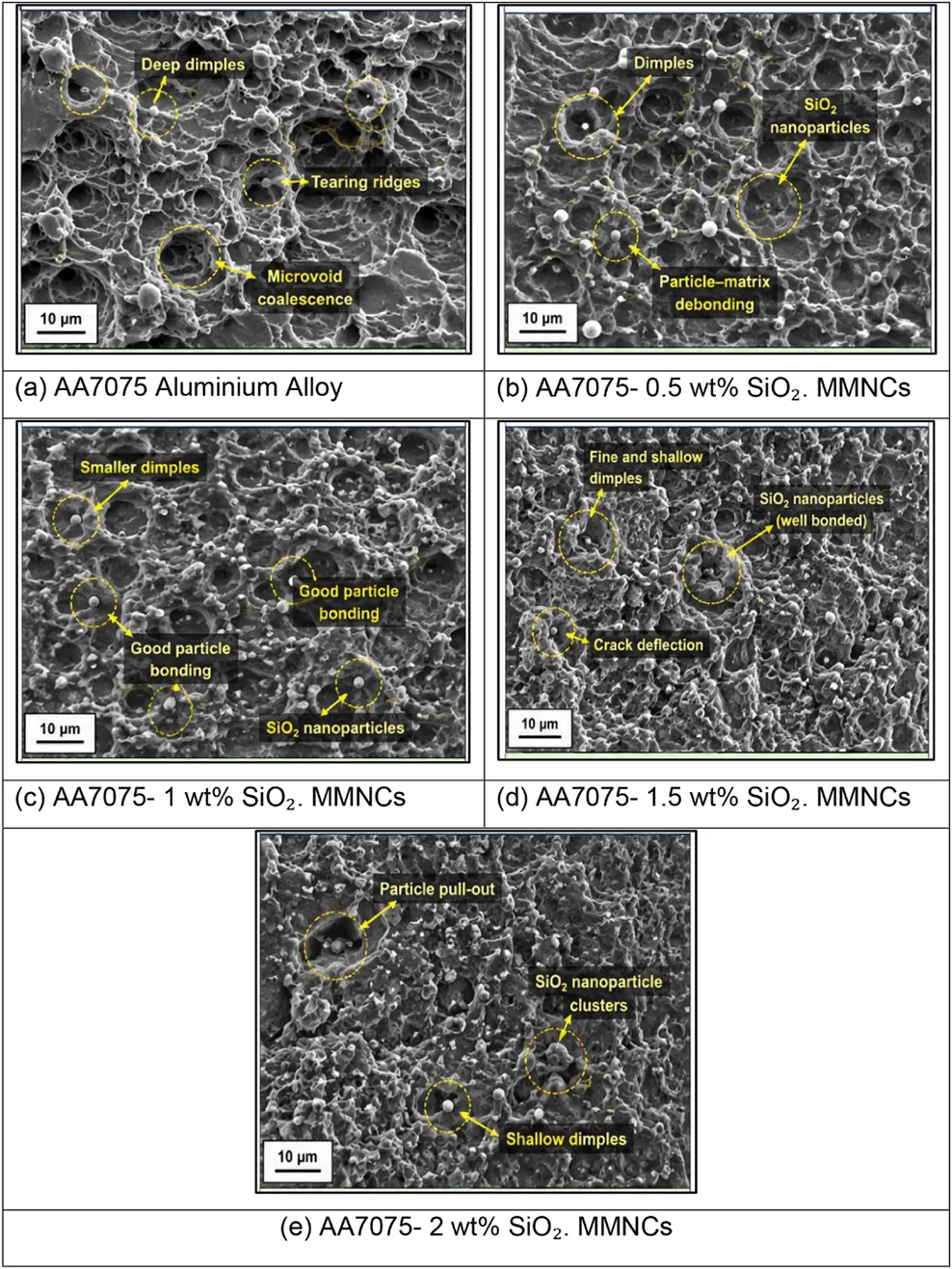
SEM micrographs of the tensile-fractured surfaces of the fabricated MMNCs: (a) AA7075, (b) AA7075–0.5 wt % SiO_2_, (c) AA7075–1.0 wt % SiO_2_, (d) AA7075–1.5 wt % SiO_2_, and (e) AA7075–2.0 wt % SiO_2_.

TEM/SAED characterization: the AA7075 alloy and AA7075–SiO_2_ composite specimens were mechanically ground and polished and then further thinned to obtain electron-transparent areas. Circular discs approximately 3 mm in diameter were prepared for TEM observation. The specimens were examined using a JEOL JEM-2100F field-emission transmission electron microscope operated at 200 kV. SAED patterns were obtained from representative regions of the Al matrix and SiO_2_-containing areas. Figure 10 shows the TEM micrographs and corresponding SAED patterns of AA7075 and the composites reinforced with 0.5, 1.0, 1.5, and 2.0 wt % SiO_2_. The unreinforced AA7075 shows relatively coarse grains with well-defined grain boundaries. The matrix is seen to be finer at 0.5 wt % SiO_2_, which indicates that the dispersed nanoparticles are involved in the nucleation and growth of Al grains during solidification. Further addition of 1.0 wt % SiO_2_ results in a more refined matrix and an improved distribution of nanoscale features. The 1.5 wt % SiO_2_ composite retains the refined structure, although some localized particle-rich regions can be identified. These regions are more obvious at 2.0 wt % SiO_2_, suggesting the formation of localized nanoparticle agglomerates at the higher reinforcement level. The SAED patterns show the concentric circular rings, which correspond to the (111), (200), (220), and (311) planes of the FCC Al matrix, signifying the polycrystalline nature of the regions studied.^67^ The SAED images were aligned with the correct aspect ratio to keep the diffraction rings circular and to avoid apparent lateral distortion. Therefore, the TEM and SAED results indicate that the addition of SiO_2_ results in gradual microstructural refinement, while the excessive reinforcement promotes the localized particle clustering in the AA7075 matrix.

**Figure 10:**
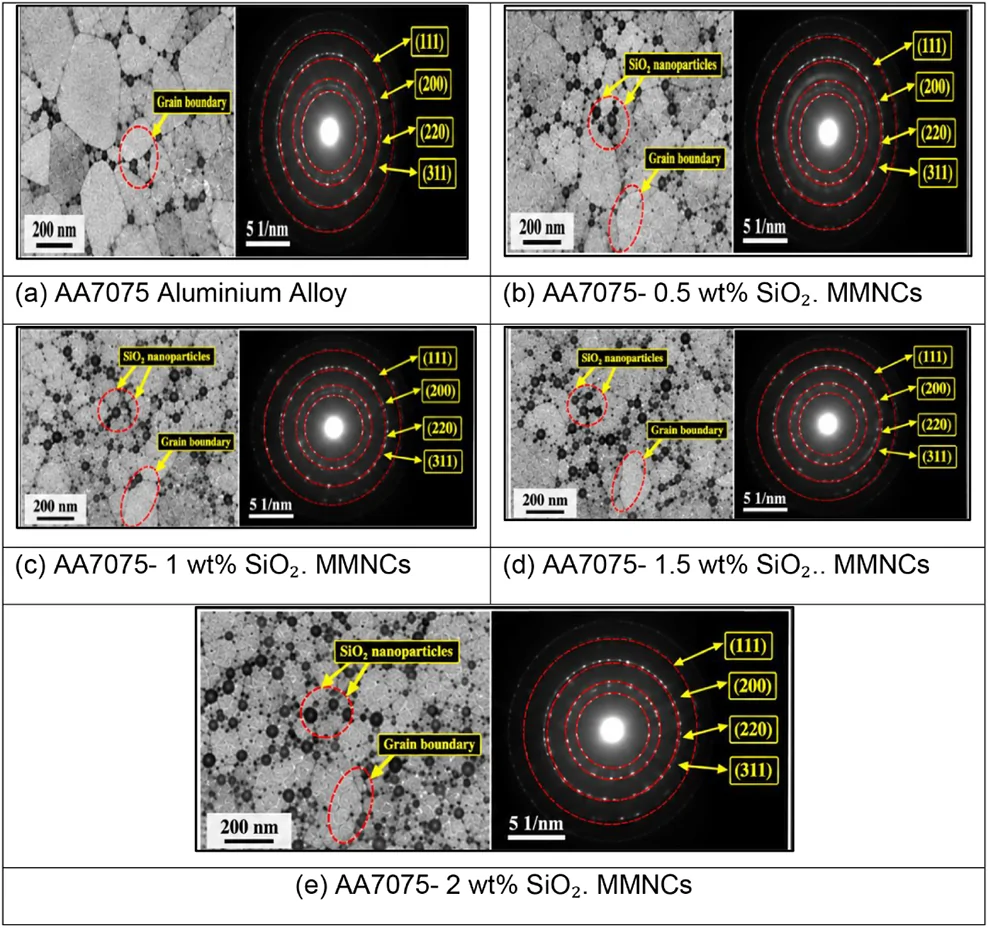
TEM images with the corresponding SAED patterns of AA7075 and AA7075–SiO_2_ composites containing 0.5, 1.0, 1.5, and 2.0 wt % SiO_2_, illustrating the changes in the Al-matrix microstructure and the characteristic diffraction rings associated with the FCC Al phase.

### 3.8 Dry-Sliding Wear Behavior of AA7075–SiO_2_ Nanocomposites

The dry sliding wear behavior of AA7075 and AA7075–SiO_2_ nanocomposites has been studied using a pin-on-disk tribometer as per ASTM G99. Tests were conducted at room temperature, with a sliding speed of 3.14 m s^–1^ and a total sliding distance of 6 km under applied loads of 10, 20, 30, 40, and 50 N. For each combination of SiO_2_ reinforcement level and applied load, three independent wear tests (n = 3) were conducted under identical conditions in accordance with ASTM G99. The specific wear rate and coefficient of friction are reported as mean values with standard deviation, and the corresponding standard-deviation error bars are shown in the revised wear and coefficient-of-friction plots. All tests were performed at 3.14 m s^–1^ over a total sliding distance of 6 km at applied loads of 10, 20, 30, 40, and 50 N. The three independent measurements for each condition were used to quantify the experimental variation in the tribological response. Figure 11a–e shows the variation in specific wear rate with sliding distance. Wear increased with increasing sliding distance for all specimens, particularly at higher loads, due to higher contact stress and progressive loss of surface material. The SiO_2_-reinforced specimens generally exhibited greater resistance to surface deformation and material removal than the unreinforced AA7075 under the investigated conditions. At 6000 m, the specific wear rate generally increased with applied load, and the SiO_2_-reinforced specimens generally exhibited lower wear rates than the unreinforced AA7075 under the investigated conditions. The 0.5 wt % composite showed an exception at 30 N, where its specific wear rate was approximately 15.9% higher than that of AA7075. At the maximum applied load of 50 N, the specific wear rate decreased from approximately 6900 μm^3^ N^–1^ mm^–1^ for AA7075 to 4500 μm^3^ N^–1^ mm^–1^ for the 2.0 wt % SiO_2_ composite, corresponding to a reduction of approximately 34.8%. Among the investigated compositions, the 2.0 wt % SiO_2_ composite generally showed the lowest specific wear rate at the higher applied loads, whereas the 1.5 wt % composite exhibited the lowest value at 10 N.

**Figure 11:**
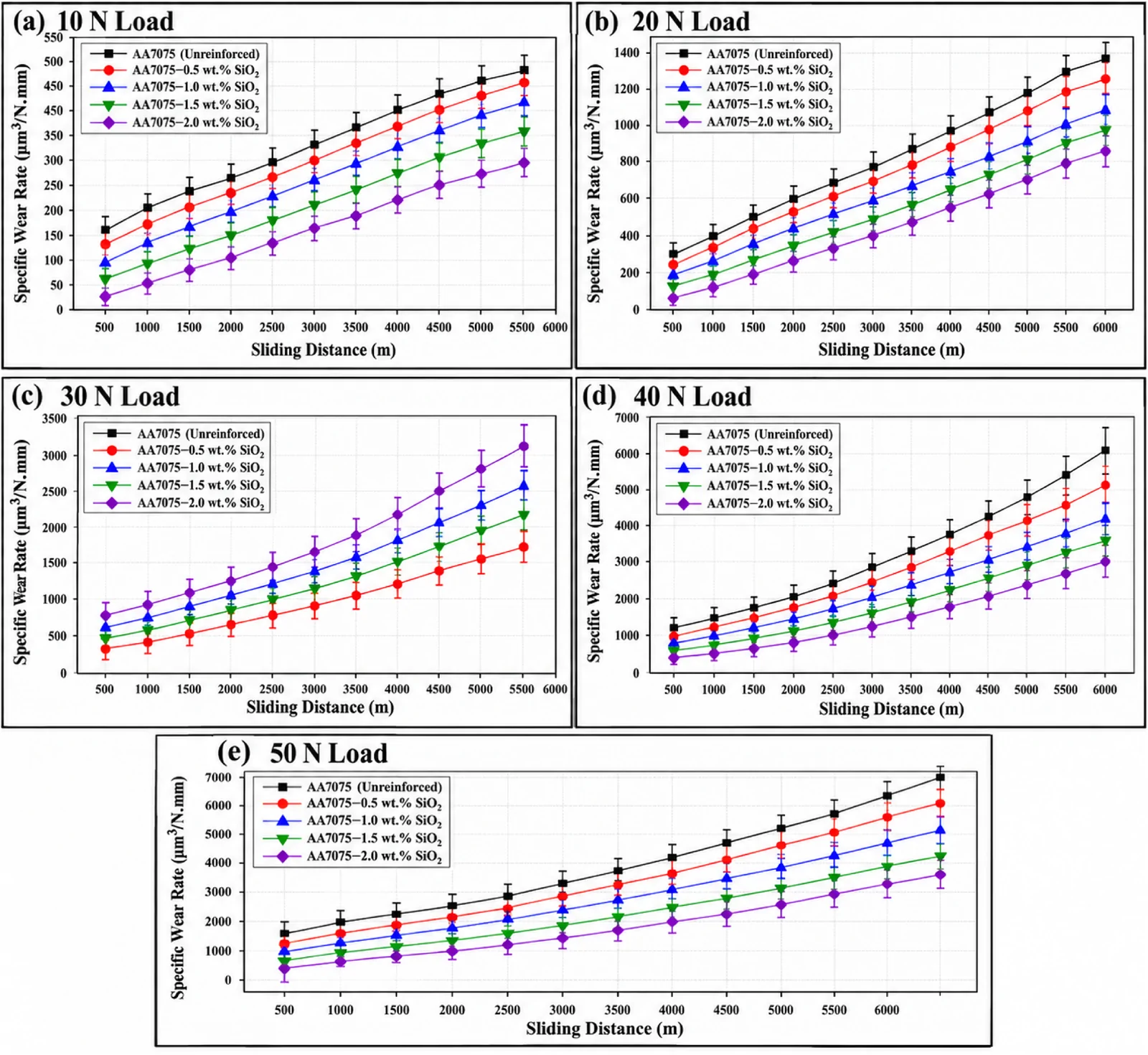
(a–e) Variation in the specific wear rate of AA7075 and SiO_2_-reinforced AA7075 nanocomposites with sliding distance at normal loads of (a) 10 N, (b) 20 N, (c) 30 N, (d) 40 N, and (e) 50 N.

The lower wear rates observed for the SiO_2_-reinforced specimens are consistent with the higher hardness and the load-supporting role of the dispersed reinforcement. The dispersed particles can also restrict localized deformation and hinder the removal of aluminum from the contact surface. The progressively better performance at higher SiO_2_ contents suggests that an adequate reinforcement concentration is required to obtain effective resistance against sliding-induced damage. Among the investigated compositions, the 2.0 wt % SiO_2_ composite generally showed the lowest specific wear rate at the higher applied loads, whereas the 1.5 wt % composite exhibited the lowest value at 10 N. The results show that the wear behavior of the developed MMNCs is governed by the synergistic effects of SiO_2_ concentration, applied load and sliding distance, and SiO_2_-reinforced specimens generally exhibited improved dry-sliding wear resistance under the investigated conditions.^63,68^

Figure 12 presents the variation in specific wear rate of the AA7075–2 wt % SiO_2_ nanocomposite with sliding distance under applied loads ranging from 10 to 50 N. For all the applied loads, the specific wear rate shows a progressive increase with increasing sliding distance, indicating the accumulation of tribological damage at the sliding interface. At a particular sliding distance, the specific wear rate increases with increasing applied load. At 6000 m, the specific wear rate increased from approximately 380 μm^3^/N·mm at 10 N to 960, 2550, 3900, and 4500 μm^3^/N·mm at 20, 30, 40, and 50 N, respectively. The wear rate increases with the applied load due to the higher contact stress and greater plastic deformation at the asperity junctions, which promote material removal and wear-debris formation.^63,69^ The increase in wear rate with sliding distance can be related to repeated deformation of the surface, breakdown of the tribolayer, accumulation of wear debris, and continuous exposure of fresh matrix material to the counterface. The SiO_2_ nanoparticles increase the load-bearing and localized deformation resistance of the AA7075 matrix, but under severe loading conditions, interfacial damage, particle pull-out, and abrasive action by detached particles can occur. Thus, the maximum wear rate was observed at 50 N and the minimum at 10 N, confirming the strong influence of applied load on the tribological response under the investigated conditions.

**Figure 12:**
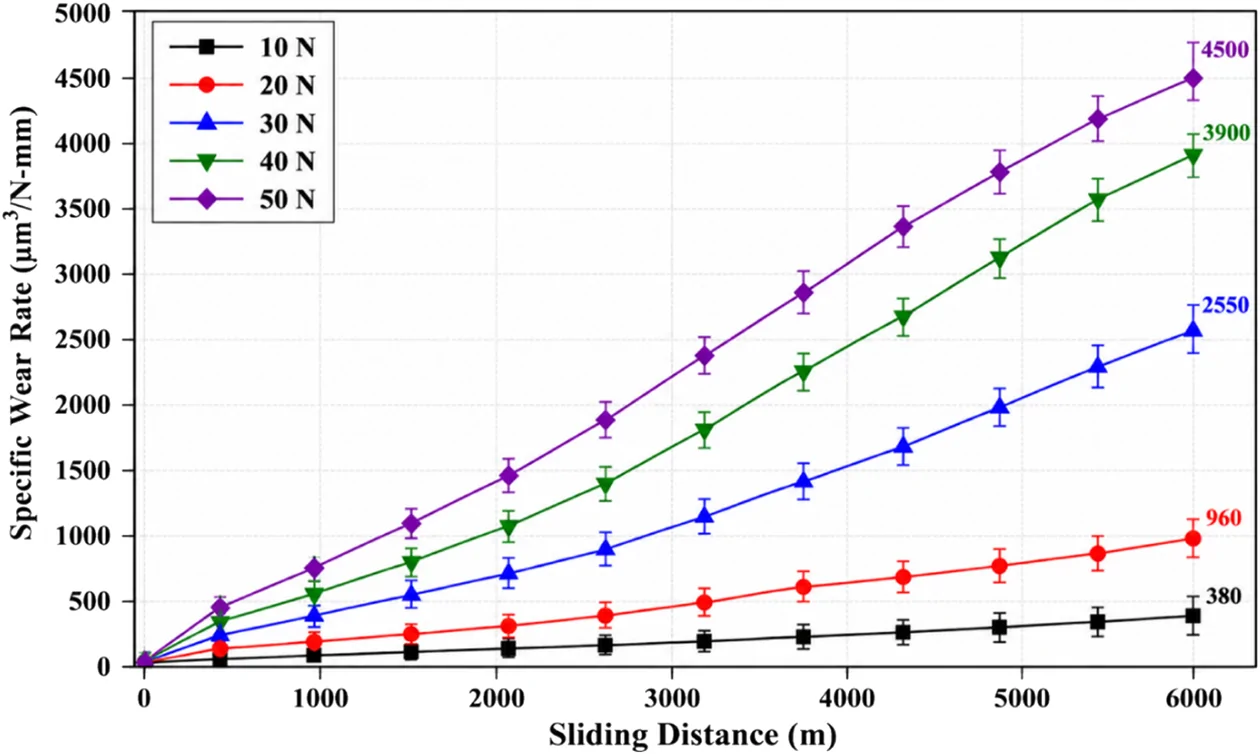
Variation of the specific wear rate of AA7075–2 wt % SiO_2_ nanocomposite with sliding distance under different applied loads.

Figure 13a–e shows that the coefficient of friction (COF) of AA7075 and AA7075–SiO_2_ nanocomposites systematically varied with sliding distance and applied normal load in the investigated range of 10–50 N. The coefficient-of-friction values reported for each load and reinforcement level represent the mean response of three independent tests (n = 3).

**Figure 13:**
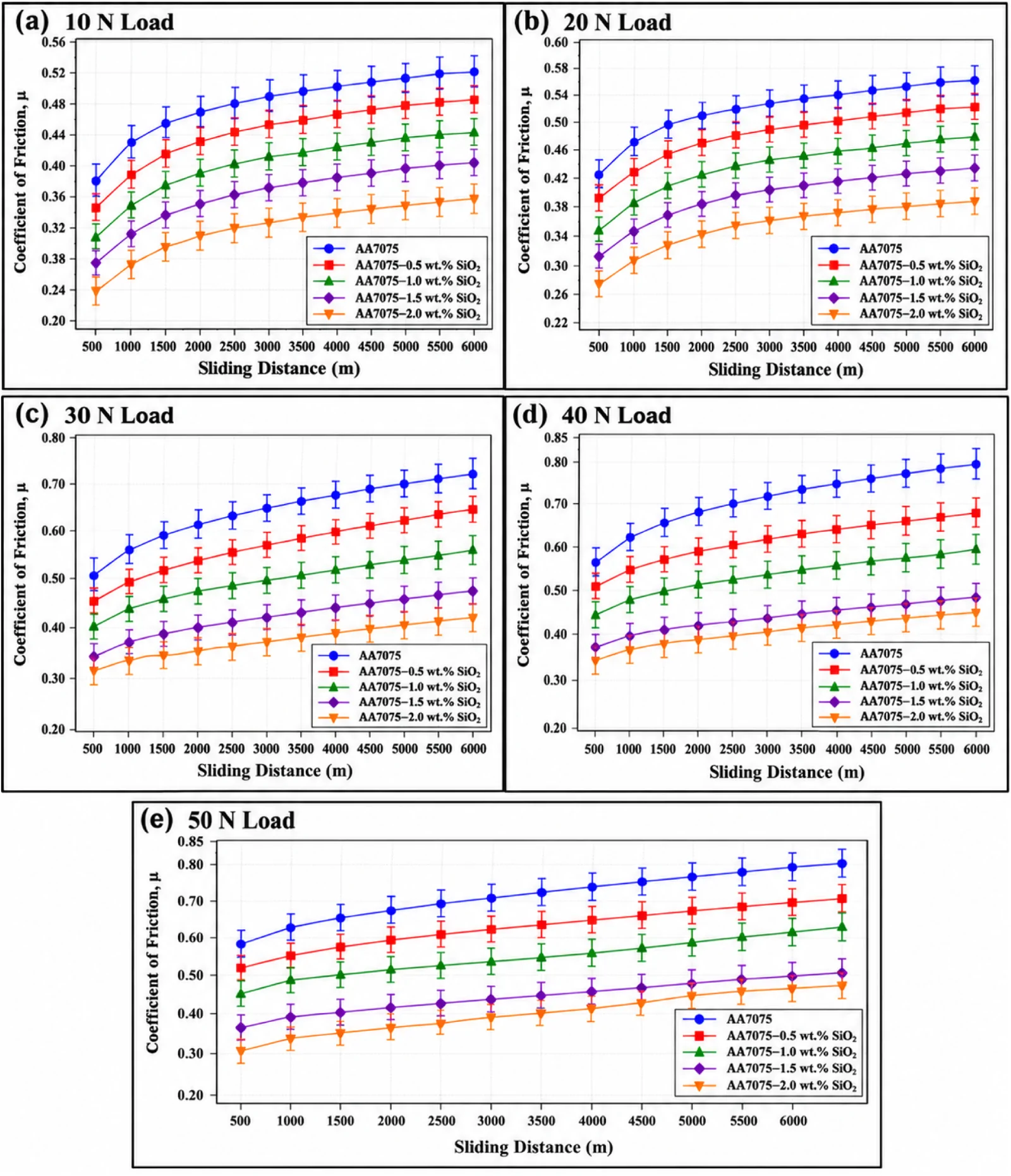
(a–e) Variation of the coefficient of friction of AA7075–SiO_2_ nanocomposites with sliding distance at different normal loads (10, 20, 30, 40, and 50 N).

Standard deviations are shown as error bars in the revised COF plots to represent the experimental scatter. The observed decrease in mean COF with increasing SiO_2_ content is therefore interpreted together with the measured variability rather than as a single-test trend. The COF values were 0.52, 0.48, 0.44, 0.40, and 0.37 at a sliding distance of 6000 m and a load of 10 N for AA7075, AA7075–0.5 wt % SiO_2_, AA7075–1.0 wt % SiO_2_, AA7075–1.5 wt % SiO_2_, and AA7075–2.0 wt % SiO_2_, respectively. At 20 N, the values were 0.56, 0.50, 0.47, 0.44, and 0.41, and at 30 N they were 0.71, 0.63, 0.56, 0.48, and 0.42. Additional increases in load resulted in COF values of 0.76, 0.67, 0.60, 0.53, and 0.46 at 40 N and 0.79, 0.70, 0.63, 0.56, and 0.50 at 50 N. The COF increased by ∼51.9%, ∼45.8%, ∼43.2%, ∼40.0%, and ∼35.1% for AA7075, AA7075–0.5 wt % SiO_2_, AA7075–1.0 wt % SiO_2_, AA7075–1.5 wt % SiO_2_, and AA7075–2.0 wt % SiO_2_, respectively, when the applied load increased from 10 to 50 N at 6000 m. The increase of the friction coefficient with load and sliding distance can be associated with a larger real contact area between the interacting surfaces, stronger interfacial shear, larger frictional heating, and more pronounced plastic deformation of surface asperities.^70^ In contrast, in the case of each load condition, the increase of the SiO_2_ fraction resulted in a decrease of the COF, showing the positive contribution of the ceramic reinforcement in controlling the frictional behavior of the AA7075 matrix. The dispersed SiO_2_ particles increase the surface hardness, restrict the plastic flow underneath the surface, and share part of the externally applied load, thus reducing the direct metallic contact at the sliding interface. During subsequent sliding, these particles may also help in the development of a relatively stable mechanically mixed or compacted tribolayer, which can diminish adhesive interactions and limit severe material removal. At 6000 m, the COF decreased from 0.52 to 0.37 at 10 N, 0.56 to 0.41 at 20 N, 0.71 to 0.42 at 30 N, 0.76 to 0.46 at 40 N, and 0.79 to 0.50 at 50 N with increasing SiO_2_ content from 0 to 2.0 wt %. These modifications are equivalent to reductions of about 28.8%, 26.8%, 40.8%, 39.5%, and 36.7%, respectively, compared to the unreinforced AA7075 alloy. The AA7075–2.0 wt % SiO_2_ exhibited the lowest friction response for all the compositions studied, while the unreinforced alloy had the highest COF. The standard deviations indicate the experimental scatter associated with the measured coefficient of friction.

Figure 14a–e shows SEM images of the worn surfaces of AA7075 and AA7075–SiO_2_ MMNCs with different reinforcement contents. The figure provides a morphological comparison of the worn surfaces as a function of SiO_2_ content. The surface morphology is significantly affected by the applied load as well as the amount of SiO_2_ reinforcement. The surface of the unreinforced AA7075 exhibits relatively minor damage (shallow grooves and small amount of detached material) at 10 N. The incorporation of SiO_2_ results in a cleaner worn surface and a gradual reduction in the extent of surface deformation.

**Figure 14:**
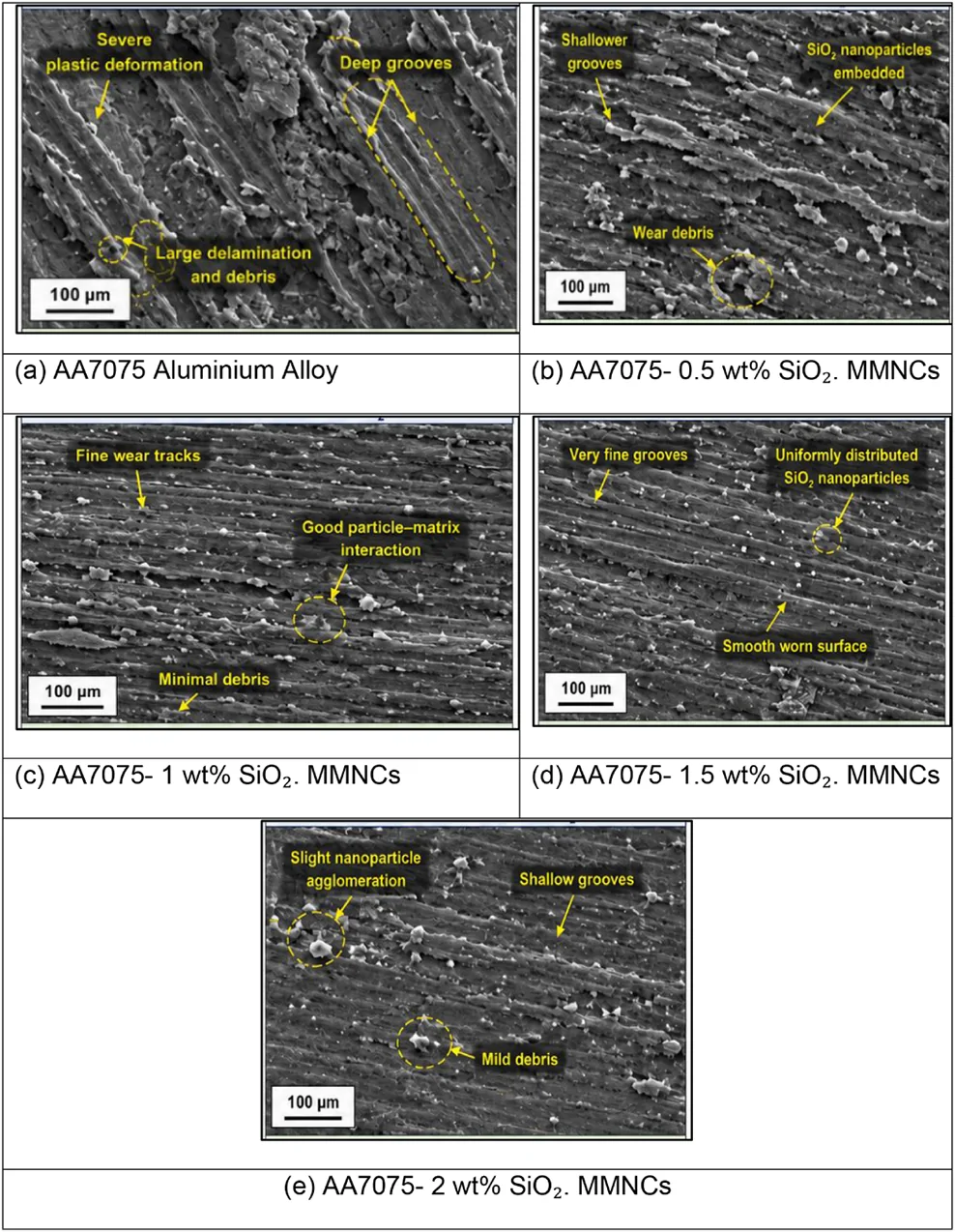
(a–e) SEM images of the worn surfaces of (a) AA7075, (b) AA7075–0.5 wt % SiO_2_, (c) AA7075–1.0 wt % SiO_2_, (d) AA7075–1.5 wt % SiO_2_, and (e) AA7075–2.0 wt % SiO_2_ nanocomposites.

When the load is increased to 20 N, the surface of the AA7075 shows deeper grooves, localized delamination, and a larger amount of wear debris, indicating more intensive material removal.^71,72^ In contrast, the SiO_2_-containing samples exhibit hardly visible grooves and less debris formation. The surface of 1.5 wt % SiO_2_ composites is rather smooth with the fine wear tracks, which indicates better resistance to surface deformation under the applied load. The unreinforced alloy shows more distinct wear tracks and more material removal at 30 N because of the higher contact pressure. The surfaces of the reinforced specimens are relatively smoother, indicating that the SiO_2_ particles support the matrix to withstand the applied sliding load. At 40 N, the worn surface of AA7075 becomes considerably rougher and contains deeper grooves, plastic deformation, delaminated regions, and accumulated wear debris. These features are less severe after SiO_2_ incorporation, particularly at the intermediate reinforcement contents. At the highest load of 50 N, the unreinforced AA7075 showed large surface damage with deep grooves and severe plastic deformation, delamination, and loose wear debris, indicating intense material removal. The addition of SiO_2_ reduces the severity of these features; however, localized particle agglomeration can be observed at 2.0 wt % SiO_2_. Such agglomerated regions may weaken the local matrix support and provide preferential locations for crack initiation or material detachment during sliding.^73^ The improved surface condition obtained with SiO_2_ addition is mainly related to the increased hardness and load-supporting ability of the reinforced matrix. The nanoparticles can also restrict localized plastic flow and reduce the extent of direct interaction between the aluminum matrix and the counterface. Consequently, the worn surfaces change from heavily damaged and deeply grooved morphologies in AA7075 to comparatively fine and stable wear tracks in the reinforced materials. The SEM observations are consistent with the measured wear behavior and show features associated with adhesive, abrasive, and delamination-type damage, with their severity varying with applied load and reinforcement content.^74^ Among the investigated compositions, 1.5 wt % SiO_2_ provides the most favorable surface morphology, characterized by fine grooves, reduced debris, and relatively uniform particle distribution. Further addition to 2.0 wt % SiO_2_ does not produce a corresponding improvement because of the tendency for local nanoparticle agglomeration. Overall, increasing the load from 10 to 50 N increases the severity of surface damage, whereas an appropriate SiO_2_ addition effectively limits groove formation, delamination, and material removal from the AA7075 matrix.

## 4 Strengthening Mechanisms in AA7075–SiO_2_ Nanocomposites

The mechanical response of metal matrix nanocomposites can be affected by the size, amount and distribution of the reinforcing particles. Fine particles that are reasonably well distributed within the matrix can impede dislocation motion, whereas coarse or agglomerated particles may introduce local stress concentrations and defects.^75^ A smaller particle size also reduces the average spacing between neighboring particles, increasing the likelihood of dislocation-particle interactions. In the present AA7075–SiO_2_ composite, the nanoscale SiO_2_ particles may contribute to strengthening through the Orowan mechanism, in which dislocations bypass the particles during deformation. The magnitude of this contribution depends on particle size, interparticle spacing, reinforcement fraction, and dispersion state.^76^ Since the present experimental results do not permit the Orowan contribution to be isolated quantitatively, it is considered a supplementary strengthening mechanism rather than a dominant one.

### 4.1 Load-Transfer Strengthening

Load transfer from the AA7075 matrix to the stiffer SiO_2_ particles can contribute to the yield-strength increase when sufficient interfacial bonding is present. The corresponding contribution was estimated using eq 1:

1 $$\sigma_{yc}=\sigma_{ym}\left[V_{r}\left(\frac{S+2}{2}\right)+V_{m}\right]$$

Where S is the reinforcement particulates’ aspect ratio, Vr is the reinforcement volume fraction, Vm is the matrix volume fraction, and σ_*ym*_ is the matrix yield strength; for spherical or equiaxed particulates, S = 1.^31,75^

### 4.2 Mechanism of Orowan Strengthening

Orowan strengthening arises when dislocations bypass finely dispersed reinforcement particles. The particles act as obstacles to dislocation motion, and the resulting increase in resistance to dislocation glide contributes to the yield strength of the nanocomposite. The difference in mechanical stiffness between the SiO_2_ particles and the AA7075 matrix influences the interaction between dislocations and the reinforcing particles. These dislocation loops, associated with Orowan strengthening, serve as obstacles that prevent dislocations from moving, thereby improving the overall strength of the composite material. The magnitude of the Orowan contribution depends on particle size, interparticle spacing, reinforcement fraction, and particle dispersion.^77,78^ The contribution of Orowan strengthening in the range that contributes to the increase in yield strength of the MMNCs is estimated as

2 $$\Delta \sigma_{\mathrm{orowan}}=\frac{0.13Gb}{\lambda }\ln\frac{D}{2b}$$

The AA7075 alloy has a “b” value of 0.286 nm and a shear modulus (G) of 24.4 GPa. The value of λ is calculated for several situations, including SiO_2_ nanoparticle reinforcement at 0.5%, 1.0%, 1.5%, and 2.0 wt % concentrations, with an average particle size of 20 nm (D). When these values are substituted into eqs 2 and 3, the calculated Orowan contribution increases with increasing SiO_2_ content, the composite yield strength rises because of Orowan strengthening. The strength increases are 9.8, 14.1, 20.1, and 26.7 MPa, respectively, considering reinforcement levels between 0.5 and 2.0 wt % of SiO_2_ nanoparticles.^46^ The Orowan calculation uses the nominal nanoparticle diameter of approximately 20 nm and therefore represents an idealized contribution based on nanoscale particle dispersion. The experimentally observed micrometer-scale particle clusters at higher SiO_2_ contents may reduce the effective Orowan strengthening contribution locally.

3 $$\lambda =D\sqrt{\left[\frac{\pi }{6V_{r}}-\frac{2}{3}\right]}$$

*G*″in the AA7075’s shear modulus and b, Burgers vector. Particle diameter average (D″), and λ″- λ″-The spacing between particles.

### 4.3 Mechanism of Dislocation Strengthening

The addition of nanoscale SiO_2_ increases the matrix–particle interfacial area and introduces a thermal-expansion mismatch between the reinforcement and AA7075. During cooling, this mismatch can generate dislocations in the surrounding matrix and contribute to strengthening. During the cooling process, thermal stresses are developed due to the thermal mismatch between SiO_2_ nanoparticles and the AA7075 matrix. These stresses arise from the thermal equilibrium at the contact temperature during the process and lead to plastic deformation in the interfacial region. The stresses decrease progressively with distance from the boundary. The TEM observations provide microstructural evidence of dislocation-related features near the reinforcement–matrix interfaces.^79,80^

4 $$\Delta \sigma_{\mathrm{CTE}}=\eta Gb\sqrt{\rho }$$

The volumetric strain of the solidified composites is associated with thermal-expansion mismatch between the alloy matrix and the SiO_2_ nanoparticle reinforcement. Here, CTE denotes the coefficient of thermal expansion. Nucleation of geometrically necessary dislocation (GND) loops near the reinforced particles in the presence of this strain. These GNDs compensate for the dislocation caused by the large fluctuation of the CTE (coefficient of thermal expansion). The strain gives rise to an increase in the composite yield strength. The strength of the MMC increases with the number of particles; thus, the GND loops are more frequent. According to the Taylor strengthening mechanism, eq 5 is used to evaluate the increase in composite yield strength due to the dislocation strengthening.^81^

5 $$\rho =\frac{12\times \Delta \alpha \times \Delta T\times Vr}{D\times b\times Vm}$$

The symbol η in the current context denotes a value that is approximately equal to 1. The shear modulus (*G*) of the alloy matrix has a particular value of 26 GPa. Conversely, the Burgers vector, denoted by the symbol *b, is a* measurement of 0.286 nm, as reported in references. The CTE produces the dislocation density (ρ), and the improvement factor fd is determined by dislocation density.^82^ With the aid of eqs 5 and 6, respectively:

6 $$f_{d}=\frac{kGb\sqrt{\rho }}{\sigma_{ym}}$$

In this framework, Δα denotes the difference between the coefficients of thermal expansion of AA7075 and SiO_2_, taken as 23.6 × 10^–6^ K^–1^ and 4.6 × 10^–6^ K^–1^, respectively. For the thermal-mismatch calculation, ΔT represents the temperature interval over which the matrix and SiO_2_ experience differential thermal contraction during cooling from the processing temperature to room temperature. The appropriate reference temperature and cooling interval should therefore be defined consistently with the temperature at which the particles are incorporated and the subsequent solidification/cooling history. By entering the *G*, *k*, *b*, ρ, Δ*T*, Δα, *Vr*, and *D* values into eqs 4 and 5, the increase in “MMCs ‘YS’” due to dislocation strengthening is found to be 63.01, 94.85, 112.03, and 138.61 MPa for the corresponding reinforcement contents of 0.5%, 1.0%, 1.5%, and 2.0 wt % of nano-SiO_2_ particles, respectively, as shown. The increase in YS is associated with the higher dislocation density estimated for the SiO_2_-reinforced composites. The creation of thermal mismatch dislocations around the SiO_2_ nanoparticles would alleviate thermal stresses at the interface between the SiO_2_ nanoparticles and matrix AA7075 during cooling from the processing temperature.^83^ The thermal-expansion mismatch can generate local residual stresses and associated dislocation structures around the SiO_2_ particles during cooling. The mechanical characteristics of the matrix and the scattered particles inside particle-reinforced MMNCs differ noticeably. The dislocation density, ρ (m^–2^), near the AA7075–SiO_2_ interfaces is estimated to increase with increasing SiO_2_ content because of the thermal-expansion mismatch between the matrix and reinforcement. During solidification and subsequent cooling, the difference in the coefficient of thermal expansion (CTE) between AA7075 and SiO_2_ produces localized residual strains in the matrix surrounding the nanoparticles. These strain incompatibilities promote the generation and accumulation of dislocations in the vicinity of the matrix–particle interfaces. The resulting increase in dislocation density enhances the dislocation-mediated strengthening contribution of the composite. As shown in Figure 15, the calculated dislocation density and corresponding strengthening contribution increase with SiO_2_ content within the analytical model.

**Figure 15:**
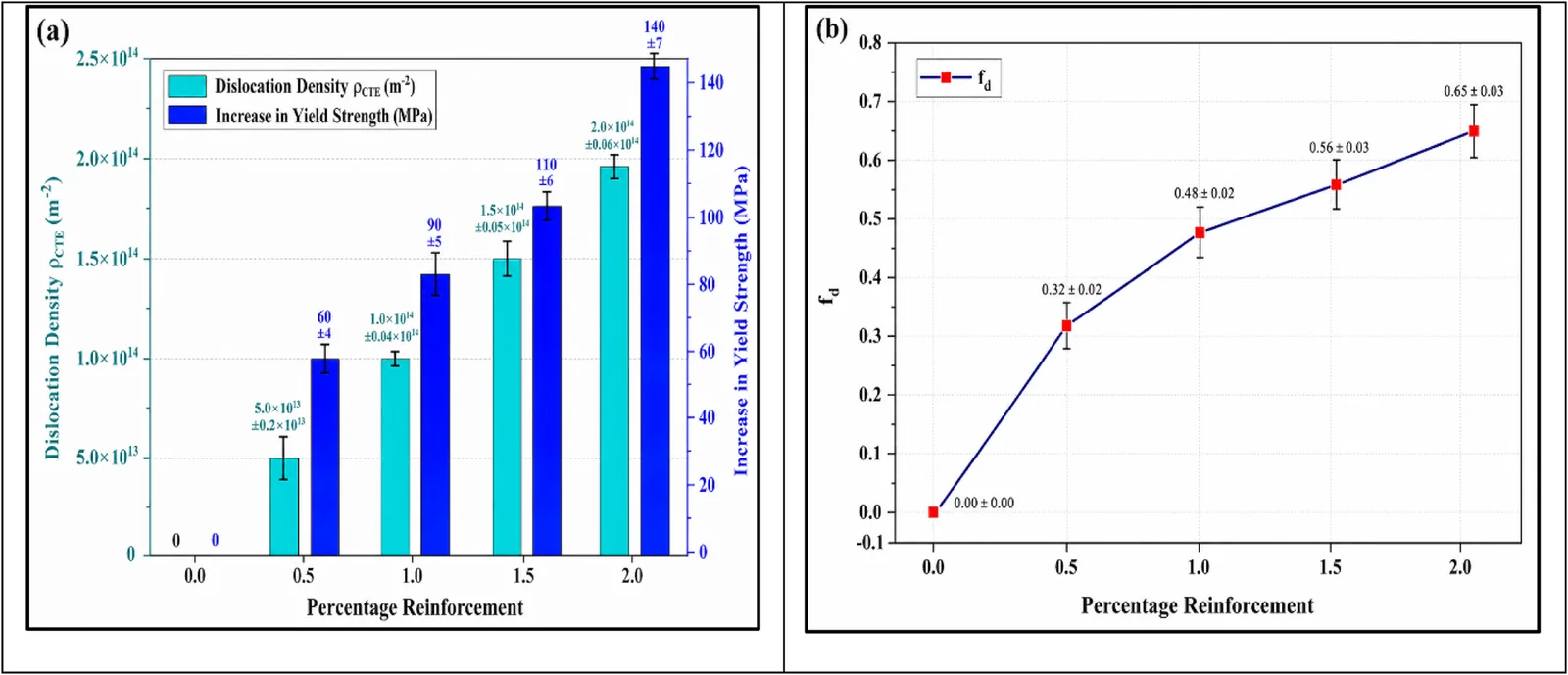
(a) Calculated dislocation density and yield-strength increase and (b) dislocation-strengthening factor as a function of SiO_2_ content.

#### 4.3.1 Strengthening with Grain Refinement

The increase in strength can partly be associated with the finer matrix grains observed after SiO_2_ addition. Grain boundaries act as barriers to dislocation motion, and their increased area in a refined microstructure can therefore contribute to yield-strength improvement. Similar microstructure–property relationships have been reported in particulate-reinforced metal matrix composites.^8^

7 $$\Delta \sigma_{Hall-Petch}=k\left(d^{-1/2}-d_{0}^{-1/2}\right)$$

The grain refinement limits the movement of dislocations, which helps to improve yield strength (YS) by increasing the grain boundary area. During solidification, the presence of SiO_2_ nanoparticles can promote heterogeneous nucleation and contribute to refinement of the matrix grain structure. Therefore, the result of the study indicates a clear relationship between the degree of restriction caused by a greater number of particles and a reduction in particle size to the nanoscale and the magnitude of YS enhancement due to grain structure refinement. Using the Hall–Petch connection, which is represented by eq 7, it is possible to predict the improvement in YS because of grain refining.^56^ In this equation, k represents the Hall–Petch coefficient of the matrix. The Hall–Petch relationship was considered to assess the possible contribution of grain refinement to the measured strengthening. The experimentally measured grain-size distributions for the SiO_2_-reinforced conditions are shown in Figure 16. The corresponding mean grain size of the unreinforced AA7075 was also determined from the metallographic analysis and is reported below for comparison. Grain size was evaluated from five separate microstructural regions for each material condition, covering approximately 120 grains per condition. The mean grain sizes were 175 ± 13 μm for unreinforced AA7075 and 143 ± 11, 105 ± 9, 95 ± 8, and 80 ± 7 μm for the composites containing 0.5, 1.0, 1.5, and 2.0 wt % SiO_2_, respectively. The results show a progressive reduction in mean grain size with increasing SiO_2_ content, indicating refinement of the AA7075 matrix. The measured grain-size variation is represented by the reported standard deviations. These measurements provide experimental evidence for a grain-boundary contribution to strengthening; however, the Hall–Petch relationship is used here only to interpret the possible contribution of grain refinement, and the calculated strengthening contributions are not treated as direct measurements of individual mechanisms.

**Figure 16:**
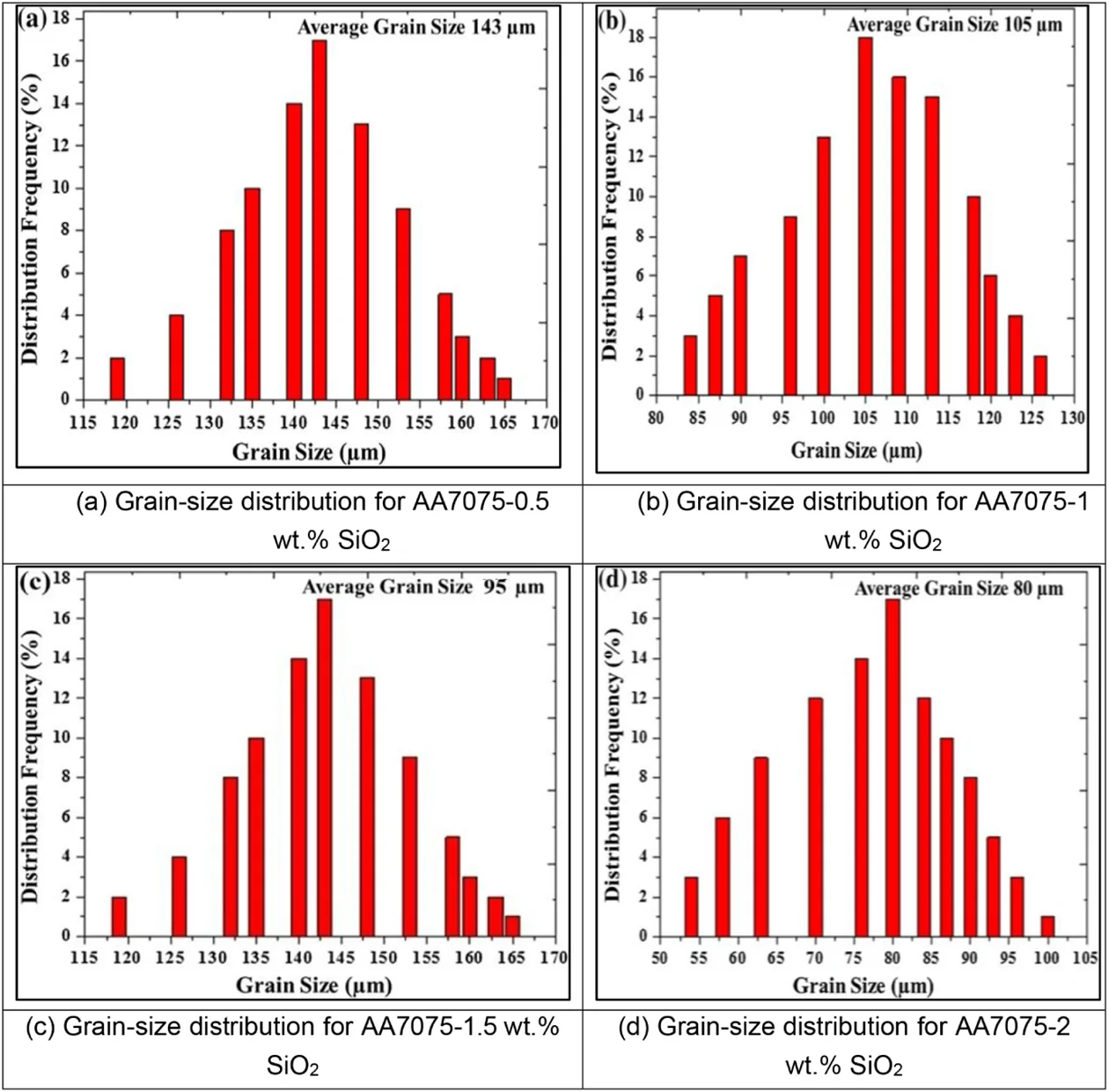
Grain-size distributions of AA7075–SiO_2_ nanocomposites containing (a) 0.5, (b) 1.0, (c) 1.5, and (d) 2.0 wt % SiO_2_.

### 4.4 Prediction and Comparison of Yield Strength

The calculated strengthening contributions were compared to assess their relative influence on the yield-strength increase. The thermal-mismatch dislocation contribution was larger than the calculated Orowan contribution within the present analytical model. Grain refinement is also observed experimentally. The measured grain sizes provide evidence for a grain-boundary contribution to strengthening; however, the Hall–Petch contribution is treated as an analytical estimate rather than as a directly measured strengthening component. The results therefore indicate that thermal-mismatch dislocation strengthening provides the largest calculated contribution among the mechanisms quantified in the present model. The effect of several micromechanical processes, which have been studied earlier, has been included in eq 8 to calculate the enhanced YS of MMCs for different reinforcing conditions.^84^

8 $$\Delta \sigma_{\mathrm{ym}}=\sigma_{\mathrm{alloy}}+\Delta \sigma_{\mathrm{Hall}-\mathrm{Petch}}\sqrt{\left(\Delta {\sigma^{2}}_{Orowan}+\Delta \sigma^{2}CTE\right)}$$

σ_alloy_ is the experimentally measured yield strength of the as-cast unreinforced AA7075 matrix used as the reference condition. Experimental testing showed the alloy yield strength (σ_alloy_) to be 114 MPa. It is worth mentioning, however, that the experimental YS values are smaller than the theoretically expected values. The reduction of YS observed is ascribed to the residual cluster of SiO_2_ nanoparticles embedded in the composite material. Perhaps the interparticle link strength inside these clusters was lower than the strong bond between the ceramic particles and the matrix, resulting in the formation of a defect that ultimately led to premature structural deterioration.

### 4.5 Assessment of Mechanisms for Strengthening

The contribution of the different strengthening mechanisms in the AA7075–SiO_2_ nanocomposites was evaluated by comparing the experimentally measured values of yield strength (YS) with the values predicted by micromechanical calculations and the load-transfer strengthening model. The difference between the calculated and experimental values was approximately 6–14%, indicating that the adopted model reproduced the experimental trend with moderate deviation. The remaining scatter could be associated with the assumptions made in the analytical models and microstructural nonuniformities such as residual particles, localized porosity, variations in interfacial bonding, and nanoparticle agglomeration. The strengthening calculations presented in this section are analytical estimates rather than direct experimental measurements of individual mechanisms. The calculated contributions from load transfer, Orowan strengthening, Hall–Petch grain refinement, and thermal-expansion-mismatch dislocation strengthening are based on simplified assumptions concerning particle size, particle distribution, interparticle spacing, and matrix-particle interaction. Consequently, the calculated values are used to interpret the measured yield-strength trend and should not be considered independent measurements of the individual strengthening mechanisms. As seen in Figure 17, the experimental YS increased from 164 ± 6 MPa at 0.5 wt % SiO_2_ to 224 ± 8 MPa at 1.0 wt %, 252 ± 9 MPa at 1.5 wt %, and 310 ± 10 MPa at 2.0 wt %. The micromechanical model gave 198 ± 7, 239 ± 7, 271 ± 8, and 320 ± 9 MPa for the same compositions, while the load transfer model predicted 210 ± 8, 247 ± 8, 285 ± 9, and 342 ± 11 MPa, respectively. The increasing trend in the experimental and calculated values indicates that increasing SiO_2_ content increases the resistance of the AA7075 matrix to plastic deformation under the investigated conditions. Theoretical models assume ideal particle distribution and effective interfacial load transfer, leading to higher calculated strengths than experimental values, where the synthesized composites may have localized defects and variation in reinforcement dispersion. In particular, agglomeration of particles can lead to stress concentration sites, promote crack initiation, and reduce the effectiveness of nanoparticle strengthening. From the microstructural observations, the average particle-cluster size increased from 1.083 to 3.505 μm when the SiO_2_ content varied from 0.5 to 2.0 wt %. The formation of micrometer-scale particle clusters may reduce the effectiveness of nanoscale Orowan strengthening and alter local load transfer.

**Figure 17:**
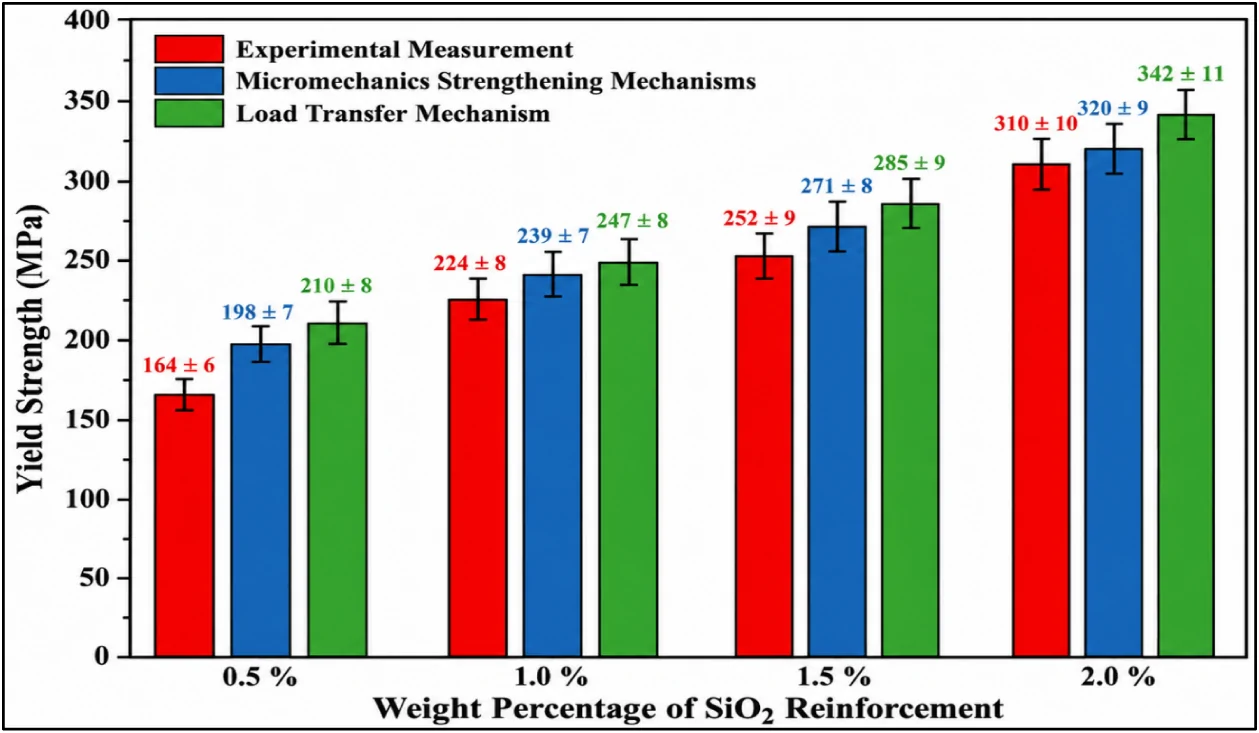
Experimental and theoretically predicted AA7075–SiO_2_ nanocomposites.

The individual strengthening contributions in Figure 18 give additional insight into the observed increase of YS. The Orowan contribution increased from 9.8 ± 0.6 MPa at 0.5 wt % SiO_2_ to 14.1 ± 0.8, 20.1 ± 1.1, and 26.7 ± 1.3 MPa at 1.0, 1.5, and 2.0 wt % SiO_2_, respectively, indicating increasing resistance to dislocation bypass with increasing nanoparticle content. The grain-refinement contribution is expected to increase with increasing SiO_2_ content as the measured mean grain size decreases; however, its quantitative contribution is treated as an analytical estimate rather than a directly measured strengthening component. The enhancement of the AA7075 matrix is associated with the observed matrix refinement and the incorporation of SiO_2_ nanoparticles during solidification. Because a nonultrasonically processed control was not included, the individual contribution of ultrasonic treatment cannot be quantified.

**Figure 18:**
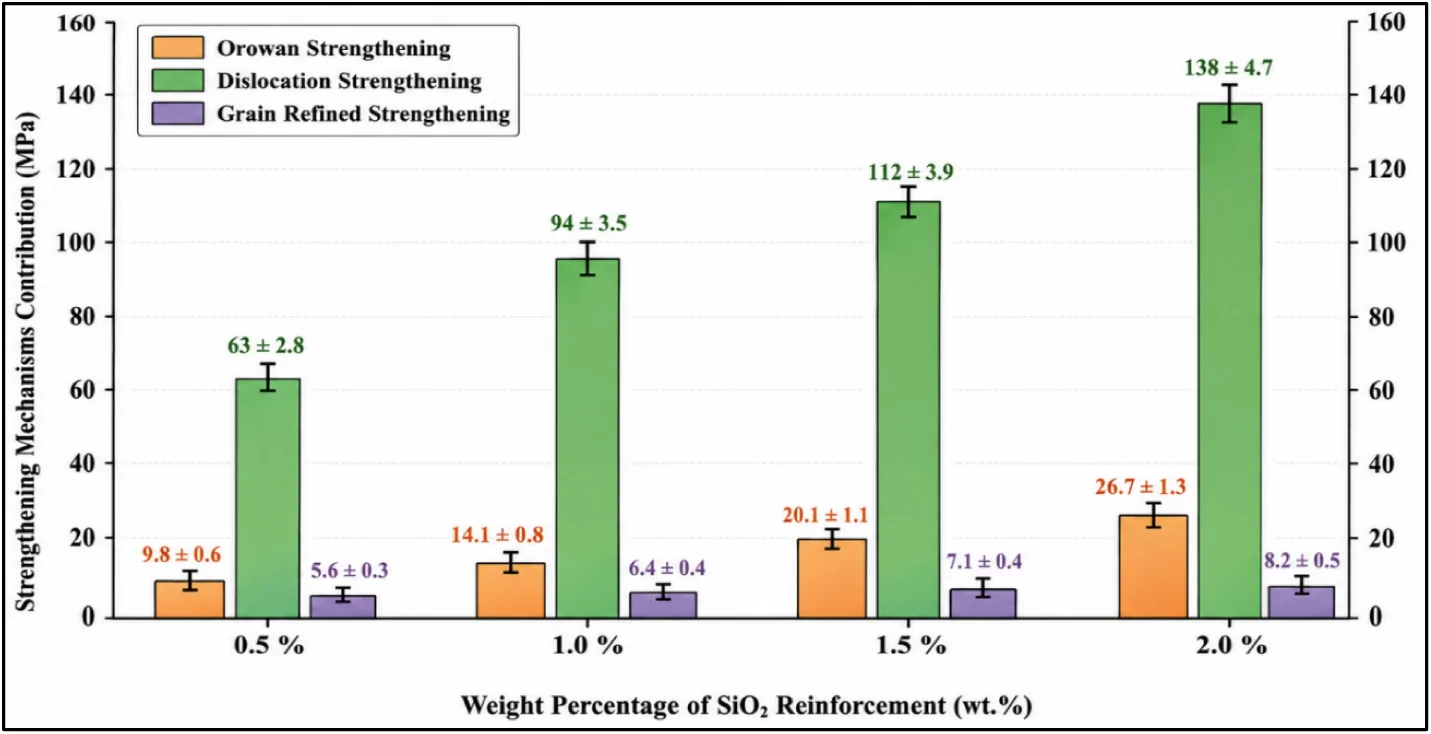
Individual contributions of Orowan, thermal-mismatch dislocation, and Hall–Petch strengthening to the yield strength of AA7075–SiO_2_ nanocomposites.

Among the mechanisms included in the quantitative analysis, thermal-mismatch dislocation strengthening gave the largest calculated contribution to the yield-strength increasing from 63 ± 2.8 MPa at 0.5 wt % SiO_2_ to 94 ± 3.5 MPa at 1.0 wt %, 112 ± 3.9 MPa at 1.5 wt %, and 138 ± 4.7 MPa at 2.0 wt % SiO_2_. The strong contribution could be due to the accumulation and limited movement of dislocations around the nanoscale reinforcement and the strain fields generated by the mismatch of the coefficients of thermal expansion between the AA7075 matrix and SiO_2_ particles.^85^ The measured strengthening response is consistent with several possible contributions, including interfacial load transfer, Orowan strengthening, matrix refinement, and thermal-mismatch-induced dislocation strengthening. The relative contribution of these mechanisms cannot be separated experimentally within the present study. In general, these contributions grow with increasing SiO_2_ content. The particle agglomeration and porosity due to casting partially compromise the achievable strengthening. Overall, the strengthening response is consistent with the combined contributions of interfacial load transfer, Orowan strengthening, matrix refinement, and thermal-mismatch-induced dislocation strengthening. The relative contribution of these mechanisms cannot be separated experimentally within the present study.

## 5 Conclusion

The present study was undertaken to investigate the effect of SiO_2_ nanoparticle addition on microstructure, tensile properties, hardness, and dry-sliding wear behavior of AA7075 fabricated by ultrasonic-assisted stir casting. The experimental observation and analytical evaluation lead to the following conclusions:

1. AA7075–SiO_2_ nanocomposites containing 0.5–2.0 wt % SiO_2_ were fabricated using the ultrasonic-assisted stir-casting route. Microstructural examination showed relatively homogeneous particle distribution and a finer matrix structure up to 1.5 wt % SiO_2_, whereas localized particle-rich regions and agglomeration became more evident at 2.0 wt % SiO_2_. Because a conventional stir-casting control without ultrasonic treatment was not included, the independent contribution of ultrasonic processing cannot be separated from the effect of SiO_2_ addition.
2. Increasing SiO_2_ content across the investigated range was associated with higher tensile strength and hardness. The UTS increased from 190 ± 15 MPa for the unreinforced alloy to 255 ± 22, 310 ± 10, 360 ± 22, and 385 ± 12 MPa for 0.5, 1.0, 1.5, and 2.0 wt % SiO_2_, respectively. Microhardness increased from 86 ± 3 to 164 ± 12 VHV. The corresponding decrease in elongation from 11.2 ± 1.5% to 8.6 ± 0.8% reflects the inherent strength-ductility trade-off associated with particulate reinforcement.
3. The measured strengthening response is consistent with the combined effects of interfacial load transfer, Orowan strengthening, matrix refinement, and thermal-expansion-mismatch-induced dislocation strengthening. Among the mechanisms quantified in the analytical model, thermal-mismatch dislocation strengthening gave the largest calculated contribution. These calculated contributions are model-based estimates rather than direct measurements of individual strengthening mechanisms, and their deviation from the experimental response reflects the simplifying assumptions of the models and the observed microstructural heterogeneity.
4. The specific wear rate generally increased with applied load and sliding distance, whereas SiO_2_-reinforced specimens showed lower wear rates than unreinforced AA7075 under most investigated conditions. At 50 N and 6000 m, the specific wear rate decreased from approximately 6900 to 4500 μm^3^ N^–1^ mm^–1^ for the 2.0 wt % SiO_2_ composite, corresponding to a reduction of approximately 34.8%. The coefficient of friction also generally decreased with increasing SiO_2_ content under the investigated conditions.
5. The SiO_2_-reinforced composites generally exhibited less severe surface grooving, delamination, plastic deformation, and wear-debris accumulation than the unreinforced alloy. These morphological observations are consistent with the measured improvement in wear response and the higher hardness of the reinforced materials.
6. Among the compositions investigated, 2.0 wt % SiO_2_ gave the highest measured tensile strength and hardness and generally showed the lowest wear rate at the higher applied loads, whereas 1.5 wt % SiO_2_ exhibited relatively more uniform particle distribution. The results indicate that increasing reinforcement content does not necessarily improve particle homogeneity, highlighting the importance of controlling nanoparticle dispersion and restricting agglomeration to optimize the simultaneous mechanical and tribological performance of AA7075–SiO_2_ nanocomposites.

The conclusions of this study are restricted to as-cast AA7075–SiO_2_ materials evaluated at room temperature under the specified tensile and dry-sliding conditions. Fatigue, corrosion, thermal stability, heat-treatment response, elevated-temperature wear, and long-duration tribological behavior were outside the scope of the present work. The strengthening analysis is based on simplified analytical models and should therefore be interpreted as an estimate of the relative contributions rather than as direct measurement of individual mechanisms. In addition, because a conventional stir-casting control without ultrasonic treatment was not included, the independent contribution of ultrasonic processing cannot be separated from the effect of SiO_2_ addition. Future work should examine a wider reinforcement range, independently evaluate processing parameters, and assess heat-treated conditions, corrosion, elevated-temperature wear, and longer sliding durations.

## Data Availability

The data supporting the findings of this study are available from the corresponding author upon reasonable request.
